# *rtfA* controls development, secondary metabolism, and virulence in *Aspergillus fumigatus*

**DOI:** 10.1371/journal.pone.0176702

**Published:** 2017-04-28

**Authors:** Ryan R. Myers, Timothy D. Smith, Sherine F. Elsawa, Olivier Puel, Souraia Tadrist, Ana M. Calvo

**Affiliations:** 1 Department of Biological Sciences, Northern Illinois University, Dekalb, Illinois, United States of America; 2 Toxalim (Research Centre in Food Toxicology), Université de Toulouse, INRA, ENVT, INP-Purpan, UPS, Toulouse, France; Woosuk University, REPUBLIC OF KOREA

## Abstract

Invasive aspergillosis by *Aspergillus fumigatus* is a leading cause of infection-related mortality in immune-compromised patients. In order to discover potential genetic targets to control *A*. *fumigatus* infections we characterized *rtfA*, a gene encoding a putative RNA polymerase II transcription elongation factor-like protein. Our recent work has shown that the *rtfA* ortholog in the model fungus *Aspergillus nidulans* regulates morphogenesis and secondary metabolism. The present study on the opportunistic pathogen *A*. *fumigatus rtfA* gene revealed that this gene influences fungal growth and conidiation, as well as production of the secondary metabolites tryptoquivaline F, pseurotin A, fumiquinazoline C, festuclavine, and fumigaclavines A, B and C. Additionally, *rtfA* influences protease activity levels, the sensitivity to oxidative stress and adhesion capacity, all factors important in pathogenicity. Furthermore, *rtfA* was shown to be indispensable for normal virulence using *Galleria mellonella* as well as murine infection model systems.

## Introduction

*Aspergillus fumigatus* has transitioned from being considered a saprophytic fungus of minor interest to recognition as one of the most important fungal pathogens, causing up to 90% of systemic *Aspergillus* infections [1]. This fungus produces spores, denominated conidia or conidiospores, which are ubiquitously present in the air and are capable of reaching the lung alveoli, where the infection is initiated [2, 3]. The spores of *A*. *fumigatus* range between 2–3 μm in diameter compared to the 3.5–8 μm diameter of other Aspergilli [2, 4]. The smaller size allows the conidia to evade capture by cilia and the mucosa [2]. Immunocompromised individuals are the main at-risk group for this fungal infection. This group includes patients who have received transplants, cancer patients undergoing chemotherapy and those with hematological malignancies or AIDS [5–10].

*Aspergillus fumigatus* infections can result in aspergillomas; mycelial balls established in pre-existing lung cavities. Patients with aspergillomas are usually asymptomatic and are only diagnosed when chest radiographs are taken for other medical complications [11]. In addition, there are several types of allergic pulmonary diseases caused by *Aspergillus* species. The most severe of these is Allergic Bronchopulmonary Aspergillosis (ABPA). This pulmonary disease occurs when *A*. *fumigatus* colonizes the bronchi in patients already affected by asthma or cystic fibrosis [12–14]. The complications incurred in this disease state range from exacerbation of asthma to fatal destruction of the lungs [15, 16].

However, the most severe condition caused by *A*. *fumigatus* infection is invasive aspergillosis (IA), with a mortality rate of 40–90% in immunocompromised patients [1, 11, 17–19]. Specific diagnostic methods as well as treatments are still limited, and in many cases not effective [20]. The prevalence and often-fatal effects of IA demands further investigation to gain insight into *A*. *fumigatus* infection traits that could provide the basis for the development of novel successful therapeutic approaches, for instance, *A*. *fumigatus* virulence factors including secondary metabolites, proteases, or conidial cell components [21].

The present study includes the characterization of the *rtf1* putative ortholog in *A*. *fumigatus*. An *rtf1* homolog has been described by Stolinski and collaborators in *Saccharomyces cerevisiae* [22]. It encodes a protein that functions as an RNA polymerase II transcription elongation factor. In that study, *rtf1* was described to have a role in transcription initiation by regulating the binding of TATA box-binding protein (TBP) to the TATA element of the promoter [22]. Subsequent studies have assigned additional functions for *rtf1* in yeast, including chromatin modifications. The Rtf1 protein has been shown to regulate the mono-ubiquitination of histone H2B at lysine 123 [23], as well as being key to recruit the chromatin remodeler Chd1 [24]. Further studies in both yeast and humans indicated that Rtf1 has chromatin-binding capabilities [24, 25]. Homologs of *rtf1* have been found in many organisms. For instance, in the zebra fish, *Danio rerio*, mutations in this gene cause improper organ development of the neural crest. Also, in mice, *rtf1* is required for proper embryonic stem cell identity [26, 27].

Previously, our laboratory characterized the *rtf1* ortholog in the model filamentous fungus *Aspergillus nidulans*, where this gene regulates asexual and sexual development as well as secondary metabolism, including the production of the sterigmatocystin toxin [28]. In addition, our recent study of an *rtfA* homolog in *Aspergillus flavus* also revealed that a loss of *rtfA* results in a reduction in conidial production and sclerotial formation, accompanied by a reduction in the synthesis of the carcinogenic compound aflatoxin B1 [29]. *rtfA* is conserved in numerous fungal species, including *A*. *fumigatus* [28].

Our present study revealed that *rtfA* is necessary for proper growth as well as conidiation in *A*. *fumigatus*. In addition, secondary metabolite production was also influenced by *rtfA*, specifically production of festuclavine, fumigiclavines A, B, and C, pseurotin A, fumiquinazoline C, and tryptoquivaline F. Concomitantly, the expression of *fgaPT2*, *psoA*, and *fmqA* was also influenced by *rtfA*. Furthermore, *rtfA* was indispensable for full pathogenicity in *Galleria mellonella* and murine infection models. Other virulence factors, such as protease activity and resistance to oxidative stress were also reduced in the absence of *rtfA* in this opportunistic human pathogen.

## Methods and materials

### Generation of *rtfA* deletion strain

To construct the *rtfA* deletion strain (Δ*rtfA*), a deletion cassette was generated using fusion PCR as previously described by Szewczyk and collaborators [30]. The *rtfA* 5’ and 3’ UTRs were amplified from *A*. *fumigatus* genomic DNA using primers AfumRM3_5f and AfumRM3_5r and primers AfumRM3_3f and AfumRM3_3r, respectively (primers used in this study are included in S1 Table). *A*. *parasiticus pyrG* (2645 bp) selection marker was amplified from *A*. *parasiticus* genomic DNA with primers AparapyrG_fumRM3_f and AparapyrG_fumRM3_r. The 5’ and 3’ UTRs were fused to *A*. *parasiticus pyrG* using primers AfumRM35_nested and AfumRM33_nested. *Aspergillus fumigatus* CEA17 strain was transformed with the deletion cassette to replace the *rtfA* coding region with *pyrG*. Mutant colonies were grown on solid Glucose Minimal Medium (GMM) at 37°C. Genomic DNA from selected transformants was extracted for diagnosis. Deletion of *rtfA* was confirmed by Southern blot analysis. Quantitative reverse transcriptase PCR (qRT-PCR) was also used to confirm the absence of *rtfA* expression. The *rtfA* deletion strain was named TRRM4 (S2 Table).

### Generation of a complementation strain

Creation of the *rtfA* complementation strain, TRRM5, began by generating a complementation plasmid, pRRM3. A 5750 bp fragment that included *rtfA* and the flanking UTRs was amplified from CEA10 using primers AfRM3_compFNot1 and AfRM3_compRAsc1 (S1 Table), which was then ligated into pJET cloning vector (ThermoFisher, Waltham, MA). Then the insert was released by HindIII digestions and ligated to pTDS3 [31], previously digested with the same enzyme. pTDS3 vector contains a pyrithiamine resistance selection marker. The resulting vector was denominated pRRM3. TRRM4 was transformed with pRRM3, resulting in the complementation strain TRRM5 (S2 Table). This strain was confirmed by diagnostic PCR using primers AfumRM3_Oef and AfumRM3_Oer.

### Generation of an overexpression strain

A strain overexpressing *rtfA* was generated by transforming *A*. *fumigatus* CEA17 with the pRRM2 plasmid. To generate pRRM2, the *rtfA* coding sequence was first amplified from *A*. *fumigatus* CEA10 using primers AfumRM3_Oef and AfumRM3_Oer (S1 Table), which were engineered with AscI and NotI restriction sites, respectively. The *rtfA* PCR product was digested with AscI and NotI and ligated to pTDS1 [31], previously digested with the same enzymes. The resulting plasmid was named pRRM2. It contains the *A*. *fumigatus pyrG* selection marker and a *gpdA* promoter to induce constitutive expression. Transformants were screened by PCR using primers gdpApromoF and AfumRM3_Oer. The overexpression strain was named TRRM2 (S2 Table).

### Generation of a heterologous complementation strain

The *Saccharomyces cerevisiae RTF1* coding sequence was PCR amplified from genomic DNA from the yeast strain Y2HGold (Clontech, Mountain View, CA) using primers Scer_rtf1_Afum_rtfA_f and Scer_rtf1_Afum_rtfA_r (S1 Table) and fused to the 5’ and 3’ UTRs of *A*. *fumigatus rtfA* using fusion PCR as previously described [30]. The three fragments were fused using primers Afum_rtfA_nested_f and Afum_rtfA_nested_f, engineered with *NotI* restriction sites. This cassette was digested with NotI and ligated to pTDS3 pre-digested with the same enzyme. The resulting vector was denominated pRRM4. This plasmid was then transformed into *A*. *fumigatus* TRRM3. Confirmation of the colonies obtained by this heterologous complementation was accomplished by diagnostic PCR, using primers Scer_rtf1_Afum_rtfA_f and Scer_rtf1_Afum_rtfA_r, as well as by qRT-PCR with primers Scer_rtf1_qPCR_F and Scer_rtf1_qPCR_R. The heterologous strain was denominated TRRM7 (S2 Table).

### Fluorescence microscopy

*Aspergillus fumigatus* ΔKU80 was transformed with an *rtfA*::*gfp*::*pyrG* fusion PCR cassette generated as previously described by Szewczyk [30]. Briefly, *rtfA* was amplified from CEA10 with primers AfumRM3_Oef and afumrm3r, excluding the stop codon. A *gfp*::*pyrG* fragment was PCR-amplified from plasmid p1439 using primers afumrm3gfpf and afumrm3gfpr (S1 Table). The *rtfA* 3’UTR was amplified from CEA10 with primers AfumRM3_3f and AfumRM3_3r. The three fragments were fused using primers afumrm3f and AfumRM33_Nested. The GFP-tagged transformant strain (TRRM6, S2 Table) was confirmed by PCR using primers AfumRM3_oef and AfumRM33_Nested. Conidia from TRRM6 were inoculated on the surface of coverslips immersed in Watch minimal medium [32]. The cultures were incubated for 16 h at 37°C. Localization of RtfA was visualized by observing green fluorescence (GF) using a Nikon Eclipe E600 microscope with a 60x immersion objective, Nomarski optics, and fluorochromes for green fluorescence detection (excitation, 470; emission, 525). GF images as well as differential interference contrast (DIC) and DAPI (4′,6-diamidino-2-phenylindole) images to indicate nuclear localization were obtained. Micrographs were captured with a Hamamatsu ORCA-ER digital camera processed by Hamamatsu HC Image software.

### Gene expression analysis

Total RNA was extracted from lyophilized mycelium using Trizol (Invitrogen, Waltham, MA), following the manufacturer’s instructions. Gene expression was evaluated by qRT-PCR. For qRT-PCR, 2 μg of total RNA was treated with RQ1 RNase-Free DNase (Promega, Fitchburg, WI). cDNA was synthesized with Moloney murine leukemia virus (MMLV) reverse transcriptase (Promega, Madison, WI). qRT-PCR was performed with the Applied Biosystems 7000 Real-Time PCR System using SYBR green Jumpstart *Taq* Ready mix (Sigma, St. Louis, MO) for fluorescence detection. The primer pairs used for qRT-PCR are listed in S1 Table. The expression data for each gene was normalized to the *A*. *fumigatus* 18S RNA gene expression and the relative expression levels were calculated using the 2^−ΔΔCT^ method [33].

### Morphological analysis

For assessment of the effect of *rtfA* on colony growth, *A*. *fumigatus* wild type, Δ*rtfA*, complementation, and OE*rtfA* strains were point-inoculated on solid GMM and incubated at 37°C. Colony diameter was measured after 5 days. The experiment was carried out with three replicates.

To assess the role of *rtfA* in conidiation, the same set of *A*. *fumigatus* strains were inoculated in GMM medium (1x10^6^ spores ml^-1^) and incubated at 37°C as liquid stationary cultures. Fungal mycelial mats were collected at 48 h and 72 h. Samples were homogenized and spores were quantified with a hemacytometer using a Nikon Eclipse E400 microscope. Mycelial samples were also collected for RNA extraction at 48 h and 72 h. The experiment was carried out with three replicates. In addition, conidiation was also assessed on solid GMM. After 6 days, 8 mm diameter cores were collected from the cultures 0.5 cm from the center of the colony and homogenized in water. Conidia were counted with a hemacytometer using a Nikon Eclipse E400 microscope.

Conidiation was also examined in submerged conditions. Flasks containing 50 ml liquid GMM were inoculated with conidia (1x10^7^spores ml^-1^) of *A*. *fumigatus* wild type, Δ*rtfA*, complementation, and OE*rtfA* strains. Liquid shaking cultures were incubated at 37°C and 250 rpm. Fungal pellets were viewed under a Nikon Eclipse E600 microscope 72 h after inoculation, and micrographs were captured and processed by a Nikon DS-Fi1c camera and Nikon NIS-elements software. The experiment included two replicates.

### Protease activity

*A*. *fumigatus* wild type, Δ*rtfA*, complementation, and OE*rtfA* strains were point-inoculated on GMM medium (1% agar) and 5% skim milk (Difco, Sparks, MD) and incubated at 37°C in the dark. After four days, 72 mm diameter cores were collected from the cultures, blended in 30 ml distilled water and collected in 50 ml Falcon tubes. The tubes were centrifuged at 3,500 rpm at 4°C and 1 ml of supernatant was transferred to 1.5 ml microcentrifuge tubes and spun at 10,000 rpm for 10 min at 4°C. An Azo-Casein assay was performed as previously described by Reichard and collaborators [34] with slight modifications. One-hundred microliters of supernatant were mixed with 400 μl of Azocasein (Sigma, St. Louis, MO) at a concentration of 5 mg ml^-1^ dissolved in 50 mM Tris buffer (pH 7.5), 0.2 M NaCl, 5mM CaCl_2_, 0.05% Brij 35, and 0.01% sodium azide and incubated at 37°C for 90 min. One hundred and fifty microliters of 20% Trichloroacetic acid was then added to stop the reaction and the samples were left at room temperature for 30 min. The tubes were spun at 8,000 rpm for 3 min and 500 μl of the supernatant was mixed with 500 μl 1 M NaOH. Two hundred microliters from each sample was placed into a 96 well plate in duplicates and the absorbance of the released azo group was read at 436 nm using a plate reader (Epoch by Biotek). A negative control was used with sterile distilled water with azocasein.

### Secondary metabolite extraction

Plates containing 25 ml liquid GMM were inoculated with 10^6^ spores ml^-1^ of *A*. *fumigatus* wild type, Δ*rtfA*, complementation, and OE*rtfA* strains and incubated at 37°C. Supernatant was collected at 120 h (three replicates) for secondary metabolite extraction and filtered through Miracloth (Calbiochem, Billerica, MA) into 50 ml conical centrifuge tubes. Twelve milliliters were collected from each replicate. Samples were extracted with an equal amount of chloroform. The bottom layer was then transferred to a glass beaker and allowed to dry. Extracts were resuspended in 1 ml methanol and then filtered through 0.22 μm diameter pore filters into 1.5 ml microcentrifuge tubes, where they were allowed to evaporate completely.

### Liquid chromatography and mass spectrometry analysis

Sample analysis was performed using HPLC coupled to an LTQ Orbitrap XL high-resolution mass spectrometer (Thermo Fisher Scientific, Les Ulis, France). Extracts were resuspended in 400 μl methanol and 10 μL of this suspension were injected into a reversed-phase (150 mm × 2.0 mm) 5 μm Luna C18 column (Phenomenex, Torrance, CA, U.S.A.) operated at a flow rate of 0.2 mL/min. A gradient program was performed with 0.1% formic acid (phase A) and 100% acetonitrile (phase B) with the following elution gradient: 0 min 20% B, 30 min 50% B, from 35 to 45 min 90% B, from 50 to 60 20% B. HRMS acquisitions were achieved with electrospray ionization (ESI) in the positive and negative modes as follows: spray voltage +4.5 kV, capillary temperature 350°C, sheath gas (N2) flow rate 40 au (arbitrary units), auxiliary gas (N2) flow rate 6 au in the positive mode, and spray voltage −3.7 kV, capillary temperature 350°C, sheath gas (N2) flow rate 30 au, auxiliary gas (N2) flow rate 10 au in the negative mode. Full MS spectra were acquired at a resolution of 60,000 with a range of mass-to-charge ratio (m/z) set to 50−800. The identity of fungal products was confirmed by comparison either with HPLC-MS^2^ analysis of a standard compound or on the base of results obtained in Gauthier *et al*. [35] and Cano *et al*. [36].

### Cell wall tests

To assess possible alterations of the cell wall integrity due to changes in the *rtfA* locus, the wild type, Δ*rtfA*, complementation and overexpression strains were point-inoculated on 25 ml 1% agar GMM supplemented with Congo Red at concentrations of 0, 30, 40, 50, and 60 μg ml^-1^ and incubated at 37°C. Photographs were taken at 48 h and 72 h.

In a similar experiment the same strain set was point-inoculated on 25 ml 1% agar GMM supplemented with SDS at concentrations of 0, 0.005, 0.01, and 0.02% SDS and incubated at 37°C. Colony diameter measurement were taken at 48 h and 72 h.

In addition, the strains were also tested in the presence of the anti-fungal nikkomycin Z. Strains were point-inoculated in a 24-well plate on 1% agar GMM supplemented with 0, 32, or 64 μg ml^-1^ Nikkomycin Z. Plates were incubated at 37°C and photographs were taken at 48 h after inoculation.

### 6-azauracil sensitivity

The wild type, Δ*rtfA*, complementation, and overexpression strains were point-inoculated on solid (1% agar) GMM plates supplemented with 6-azauracil at concentrations of 0, 50, 100, and 300 μg ml^-1^. Plates were incubated at 37°C and colony diameters were measured at 48 h and 72 h.

### Adhesion capacity test

In order to evaluate the possible role of *rtfA* in adhesion capacity on inanimate surfaces, each strain was inoculated in 12 ml GMM at 10^5^ spores/ml, homogenized and 130 μl of these suspensions was added to each well of a 96-well plate and incubated at 37°C for 24 h, 48 h, and 72 h. After incubation the medium was removed and the mycelia were washed three times with water. Then they were stained with 130 μl 0.01% Crystal Violet in water. Staining was allowed for 20 min at room temperature, washed three times with water, allowed to dry, and then destained with 130 μl of 30% acetic acid. The absorbance was read at 560 nm on an Epoch spectrophotometer (Biotek, Winooski, VT).

### Environmental stress tests

All strains were point-inoculated on solid GMM (1% agar) and incubated at 37°C. Fungal growth was assessed as colony diameter and documented with photographs. Temperature stress tests were performed at 25°C, 30°C, 37°C, 42°C, and 45°C. Response to osmotic stress was analyzed by supplementing the medium with either 1.2 M sorbitol, 0.6 M KCl, or 1.0 M sucrose. Analysis of the *rtfA* role in response to pH stress was performed by adjusting the pH of the medium to 5, 6, 7 or 8.

To evaluate the importance of *rtfA* in the resistance to oxidative stress, the strains were point-inoculated on solid GMM plates supplemented with 0, 5, 10, 15, 20, 25, 30, or 35 μM menadione in a 24-well plate and incubated at 37°C for 48 h or 72 h. A separate experiment was done inoculating the strains (1 x 10^7^ spores ml^-1^) in liquid GMM and growing at 30°C for 24 h. One gram of mycelium was then transferred to 50 ml liquid GMM with or without 20 μM menadione and incubated at 30°C and 200 rpm in a shaking incubator. Mycelium was collected after 6 h for gene expression analysis of *cat1* and *cat2*.

### Pathogenicity analysis in the *Galleria mellonella* model

Spore suspensions of *A*. *fumigatus* wild type, Δ*rtfA*, complementation and OE*rtfA* strains were generated in 1x PBS (with 0.1% Tween). The spore suspensions were quantified using a hemacytometer and diluted in 1x PBS to a concentration of either 1x 10^5^ or 1x10^6^ spores 10 μl^-1^. The infection procedure was done as previously described by Fuchs [37]. Briefly, *Galleria mellonella* larvae (Vanderhorst Wholesale, Saint Marys, Ohio) with a weight range between 275–300 mg and lacking grey markings were selected for the experiment. Groups of 30 larvae were selected for each *A*. *fumigatus* strain. PBS injections and larvae without injections were used as controls. The larvae were injected with spores using a syringe behind their last left pro-leg. The larvae were transferred into a Petri dish (90 mm x 15 mm) and wrapped in aluminum foil. The plates were placed at 37°C in the dark. After 16 h, the larvae were checked every two hours for mortality. Mortality curves were generated using SPSS software and a log rank test was performed to generate pair-wise comparisons of the survival of the larvae infected with different strains. No ethical approval was required for this species because they are unregulated animals.

### Pathogenicity analysis in a murine model

To examine the role of *rtfA* in an *in vivo* mammalian model, female 6-week-old, outbred Swiss ICR mice (Harlan Sprague Dawley, Indianapolis, IN), weighing 24 to 27 g, were immunosuppressed (neutropenic) by subcutaneous injection of cyclophosphamide (150 mg kg^-1^ of body weight) on days −4, −1, and 3 with a single dose of cortisone acetate (200 mg kg^-1^), given subcutaneously on the day of spore inoculation. The mice were anesthetized with isoflurane inhalation on day 0. The mice were infected by intranasal inoculation, mimicking the natural route of infection. Inoculum was prepared from three strains: wild type, Δ*rtfA*, and complementation strains grown on glucose minimal medium (GMM) plates. Conidia were harvested by flooding fungal colonies with 0.85% NaCl with Tween 80, enumerated with a hemocytometer, and adjusted to a final concentration of 1x10^6^ spores ml^-1^. Sedated mice (10 mice per fungal strain) were infected by nasal instillation of 50 μl of the inoculum (day 1) and monitored three times daily for 7 days post-infection. All surviving mice were sacrificed on day 7. Cumulative mortality curves were generated, and the statistical analysis using the log rank test was utilized to perform pairwise comparisons of survival levels among the strain groups.

This study was carried out in strict accordance with the Guide for the Care and Use of Laboratory Animals of the National Research Council. The protocol was approved by the Institutional Animal Care and Use Committee of Northern Illinois University (Permit #12–0006). All efforts were made to minimize suffering. Humane euthanasia by C0_2_ inhalation was performed when mice met criteria indicating a moribund state; these endpoints include behaviors of unresponsiveness to tactile stimuli, inactivity, lethargy, staggering, anorexia and/or clinical signs of bleeding from the nose or mouth, labored breathing, agonal respirations, purulent exudate from eyes or nose, abnormally ruffled fur, or greater than 20% weight loss. The method of euthanasia by CO_2_ inhalation is consistent with recommendations of the Panel on Euthanasia of the American Veterinary Medical Association.

### Statistical analysis

All statistical analysis was completed using IBM SPSS software. Unless otherwise indicated, three replicates were used and a p value of 0.05 was used for statistical analysis. Error bars represent standard error.

## Results

### Identification of *rtfA* in *A*. *fumigatus*

Rtf1 presents the well-characterized conserved domain Plus3, which has homology to other nucleic acid binding proteins, including the PAZ domains on the endoribonucleases Dicer and Argonaute from *Drosophila melanogaster* as well as the KOW domain of the bacterial transcription elongation factor NusG; the Plus3 domain was demonstrated to bind to RNA polymerase II as well as to single-stranded DNA in humans [25]. The Plus3 domain receives its name from three conserved positively charged amino acids—two arginine residues and a serine [25]. The domain encompasses amino acids 244–343 in *S*. *cerevisiae*. A BlastP search (NCBI) using *S*. *cerevisiae* Rtf1 as query to find homologs in *A*. *fumigatus* revealed only one hit. The *rtfA* gene (Afu2g01900) consists of 2036 nucleotides located on the positive strand of chromosome 2. The *A*. *fumigatus* RtfA deduced encoded protein (accession number, XP 749325.1) is 607 amino acids long. A comparison between *S*. *cerevisiae* Rtf1 and *A*. *fumigatus* RtfA homologs showed 27% identity. *Aspergillus nidulans* RtfA also contains the conserved Plus3 domain, identified as amino acids 271–375. Comparison between RtfA from *A*. *nidulans* (AN4570) and its homolog in *A*. *fumigatus* revealed a 66% identity.

### Growth rate and conidiation are regulated by *rtfA*

**Fig 1:**
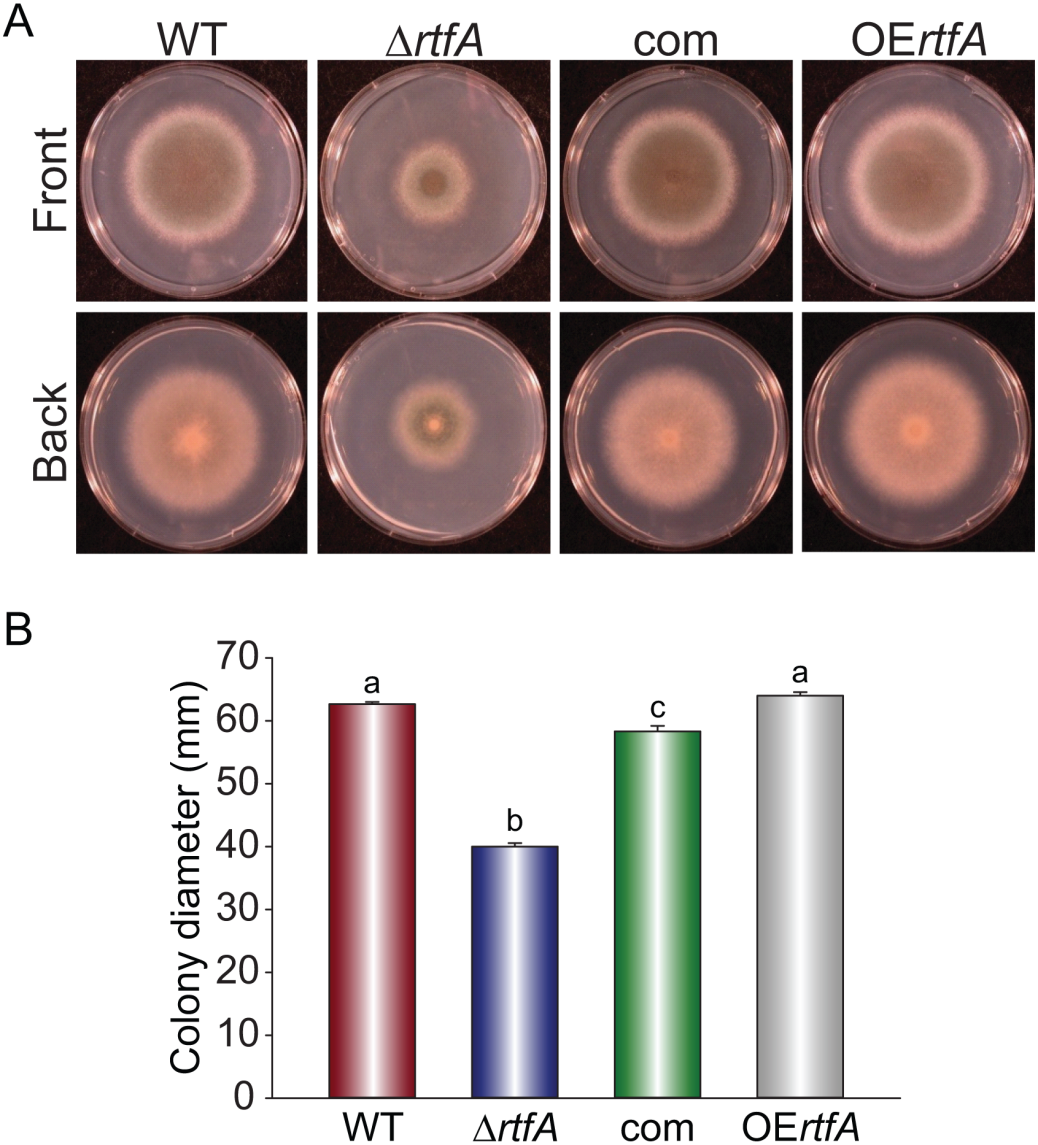
*rtfA* affects *A*. *fumigatus* colony growth. *Aspergillus fumigatus* wild type (WT), Δ*rtfA*, complementation (com), and overexpression *rtfA* (OE*rtfA*) strains were point-inoculated on GMM and incubated for 4 days at 37°C. (A) Photographs of the colonies. (B) Colony growth estimated as colony diameter. Different letters above the bars indicate significantly different values (p ≤ 0.05). Error bars represent standard error.

To determine the effects of *rtfA* on growth, conidiation, and other cellular processes, *rtfA* deletion (Δ*rtfA*), complementation, and overexpression strains were generated. The deletion strain was confirmed by Southern blot analysis (S1A Fig). Genomic DNA from both wild type and Δ*rtfA* strain was isolated and digested with *Sal I*. A 1624 kb DNA fragment corresponding to the 5’ UTR region of *rtfA* was used to generate the radioactive probe utilized in this hybridization. The presence of 5.1 and 3.7 kb bands in the Southern blot analysis confirmed the *rftA* deletion, while 5.1 and 1.6 kb bands corresponded to the wild type control (S1A Fig). With respect to the complementation strain, diagnostic PCR was used to verify the integration of the wild type allele in the Δ*rtfA* strain (S1B Fig). PCR was also utilized to verify the overexpression strains (S1C Fig). In addition, qRT-PCR was used to confirm the lack of *rtfA* expression in the Δ*rtfA* mutant under conditions that allow its transcription in the wild type and complementation control strain (S1D Fig). Forced high levels of *rtfA* transcripts in the overexpression strain (40x compared to wild type) were also confirmed by this method (S1D Fig). Examination of the colony growth rate of wild type and Δ*rtfA* strain revealed a significant decrease (37%) in colony diameter in the absence of *rtfA* compared to the wild type (Fig 1A and 1B). A green pigment appeared in the deletion mutant colony when viewed from beneath. This green pigment was absent in the isogenic wild type strain. Complementation of Δ*rtfA* with the *rtfA* wild type allele recovered wild type phenotype. Overexpression of *rtfA* did not show any effects on growth.

**Fig 2:**
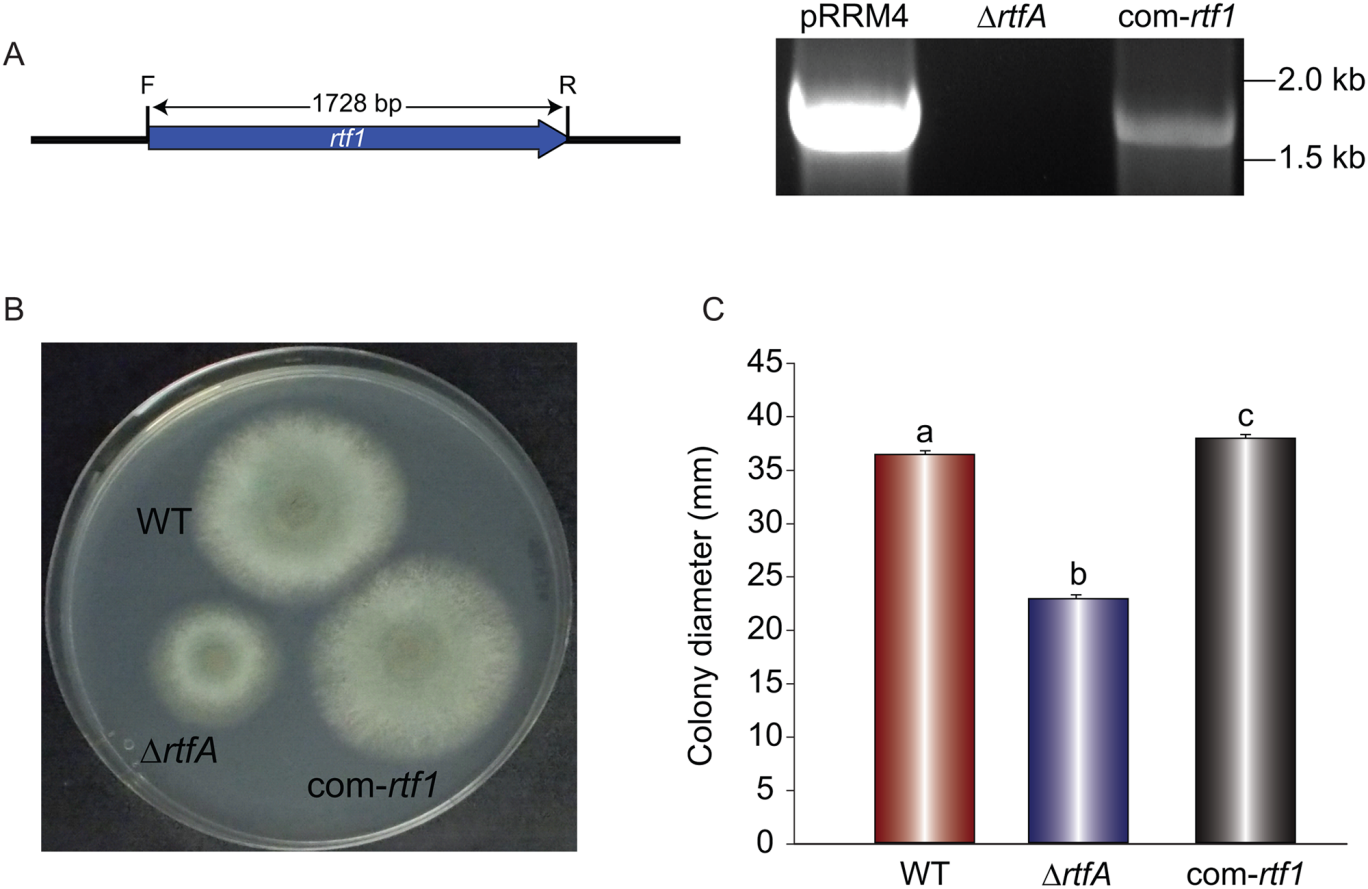
Heterologous complementation with *S*. *cerevisiae rtf1* restores wild-type phenotype in the *A*. *fumigatus rtfA* mutant. *Aspergillus fumigatus* Δ*rtfA* strain (TRRM3) was transformed with plasmid pRRM4, which contains *rtf1* from *S*. *cerevisiae*. (A) Effective complementation with *rtf1* was confirmed by diagnostic PCR analysis using primers Scer_rtf1Afum_rtfA_f and Scer_rtf1_Afum_rtfA_r. Plasmid pRRM4 was used as a positive control, yielding the expected 1.7 kb PCR product. *Aspergillus fumigatus* Δ*rtfA* strain was used as negative control. (B) *A*. *fumigatus* strains WT (CEA10), Δ*rtfA* (TRRM4), and com-*rtf1* (TRRM7) were point-inoculated on GMM and incubated at 37°C. (C) Colony diameters were measured after three days of growth. Values represent the average of three replicates. Different letters above the bars indicate significantly different values (p ≤ 0.05). Error bars represent standard error.

To examine possible functional similarity of *A*. *fumigatus rtfA* with that of *S*. *cerevisiae rtf1*, *A*. *fumigatus* Δ*rtfA* was complemented with *rtf1* from yeast (Fig 2). After successful complementation, com-*rtf1* (TRRM7) was compared to the wild type strain and the Δ*rtfA* strain. The three strains were incubated for 3 days on GMM. Our results showed that complementation with the *S*. *cerevisiae* genes rescued wild-type phenotype in *A*. *fumigatus*.

Previous studies indicated that *rtf1* is a transcription elongation factor [23, 24]. Cchemical treatment with 6-azauracil (6-AU) results in a reduction of intracellular GTP and UTP levels, which alone is not lethal, but in combination with mutations that affect transcriptional elongation, can prevent growth [38, 39]. Loss of *rtf1* in *S*. *cerevisiae* significantly affected the ability of the yeast strain to grow in the presence of 6-AU [40]. We tested the *A*. *fumigatus* Δ*rtfA* strain’s ability to grow in the presence of this chemical. Increasing concentrations of 6-AU in the medium lead to a gradual decrease in growth in all the strains assayed. Addition of this compound to the Δ*rtfA* cultures only slightly aggravated the observed colony growth reduction in *A*. *fumigatus* (S2 Fig).

**Fig 3:**
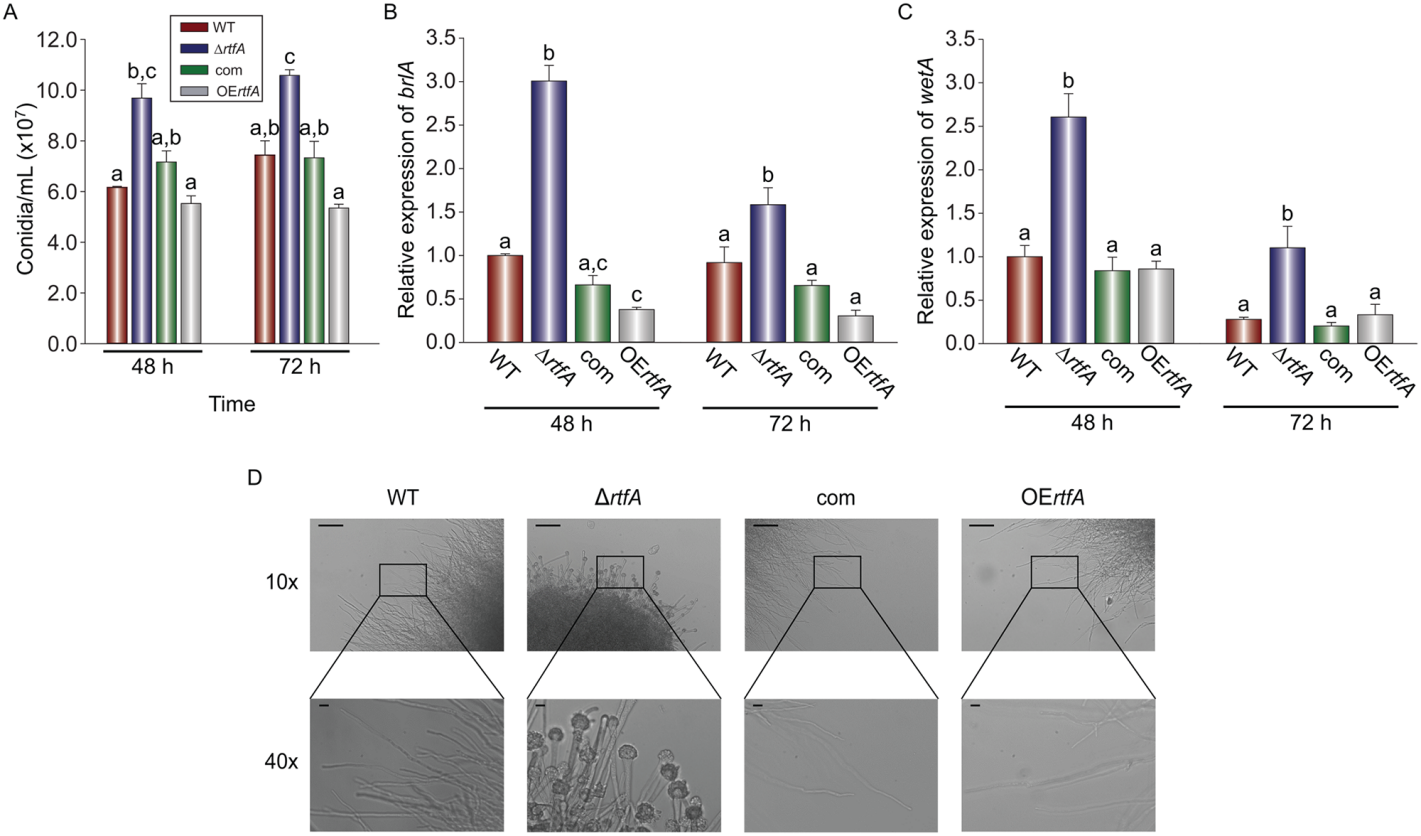
*rtfA* is a repressor of conidiation in *A*. *fumigatus*. (A) Wild type (WT), Δ*rtfA*, complementation (com), and overexpression *rtfA* (OE*rtfA*) strains were grown in liquid stationary cultures and whole mycelial mats were collected at 48 h and 72 h, homogenized, and conidia were quantified with a hemocytometer. Expression analysis of *brlA* (B) and *wetA* (C), two key genes in the conidiation regulatory pathway, at 48 h and 72 h. (D) Micrographs showing conidiation in the Δ*rtfA* strain when grown in liquid shaking cultures. Cultures were grown at 37°C for 72 h at 250 rpm. Scale bars represent 100 μm in the 10x micrographs and 10 μm in the 40x micrographs.

Airborne conidia are infectious inoculum for aspergillosis. Our previous studies indicated that production of these asexual spores was affected by *rtfA* in *A*. *nidulans* and *A*. *flavus* [28, 29]. In our current study, the role of *rtfA* in conidiation was also examined in *A*. *fumigatus*. The Δ*rtfA* strain showed a significant increase in conidial production compared to the wild type in liquid stationary cultures (Fig 3A). This hyperconidiation coincided with increased expression of two key regulatory genes in this developmental signaling pathway, *brlA* and *wetA* (Fig 3B and 3C) [41, 42]. Similarly, the *rtfA* deletion mutant also presented hyperconidiation when grown on solid medium (S3A Fig). Furthermore, conidiation was also observed in Δ*rtfA* in submerged cultures, a condition that inhibits conidiophore formation in the wild type (Fig 3D). Additionally, the Δ*rtfA* submerged culture accumulated a purple compound that was absent in the wild-type culture (S3B Fig).

### RtfA subcellular localization

Previous work in *A*. *nidulans* revealed the nuclear localization of the RtfA homolog in this model fungus [28]. To investigate whether this holds true in *A*. *fumigatus*, we generated a strain with RtfA fused to GFP. The generated strain presented wild-type phenotype. Our results indicated that *A*. *fumigatus* RtfA is also localized in nuclei, as indicated by comparison with images corresponding to DAPI staining (S4 Fig).

### *rtfA* is required for normal protease activity

**Fig 4:**
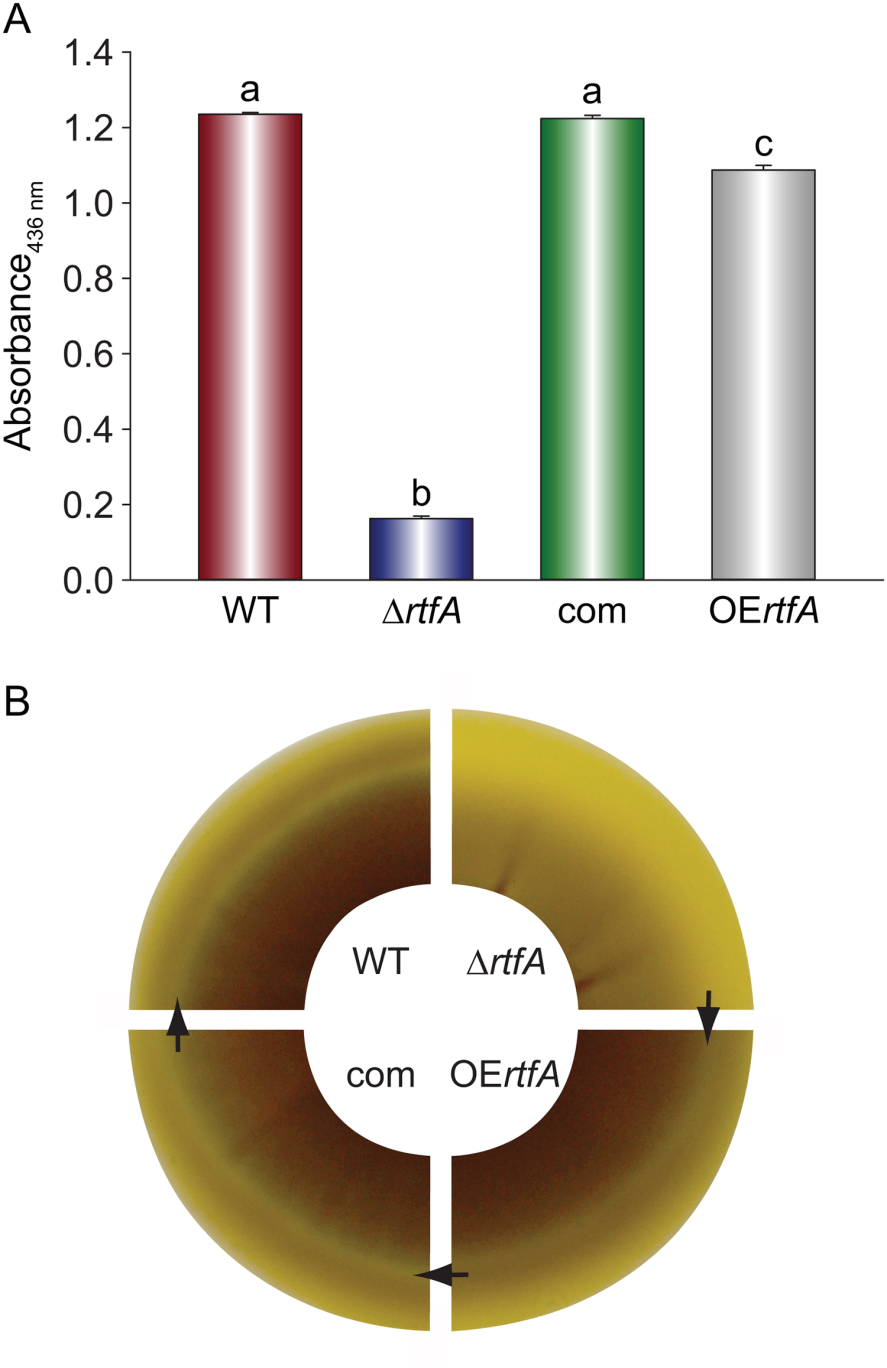
*rtfA* regulates protease activity. *Aspergillus fumigatus* wild type (WT), Δ*rtfA*, complementation (com), and overexpression *rtfA* (OE*rtfA*) strains were point-inoculated on GMM containing 5% skim milk (Difco) and incubated at 37°C. (A) Quantification of proteolytic activity is shown as measured by Azo-casein assay after four days of growth. Different letters above the bars indicate significantly different values (p ≤ 0.05). Error bars represent standard error. (B) Degradation halos (indicated with arrows) at the edge of the colonies growing on skim milk agar plates for four days.

Studies in *A*. *nidulans* have shown that *rtfA* is functionally dependent on the global regulatory gene *veA* [28]. Our previous studies on *A*. *fumigatus veA* revealed that absence of *veA* or overexpression of this gene results in a decrease in protease activity levels [21]. It is possible that *rtfA* in *A*. *fumigatus* could also influence protease activity in this fungus, which has been associated with pathogenicity in some studies [43, 44] while not in others [45–47]. Similarly to the *veA* study, our current study revealed that alteration of the *A*. *fumigatus rtfA* locus results in variations in protease activity compared to those in the wild type (Fig 4A). Specifically, deletion of the *rtfA* gene led to a significant decrease of this hydrolytic activity when the cultures were grown on 5% skim milk agar plates. The proteolytic activity in cultures can be also visualized by degradation halos forming at the edge of the colonies. The degradation halo was absent in the deletion mutant, while they were visible in the other strains assayed (Fig 4B).

### *rtfA* influences *A*. *fumigatus* oxidative stress sensitivity

**Fig 5:**
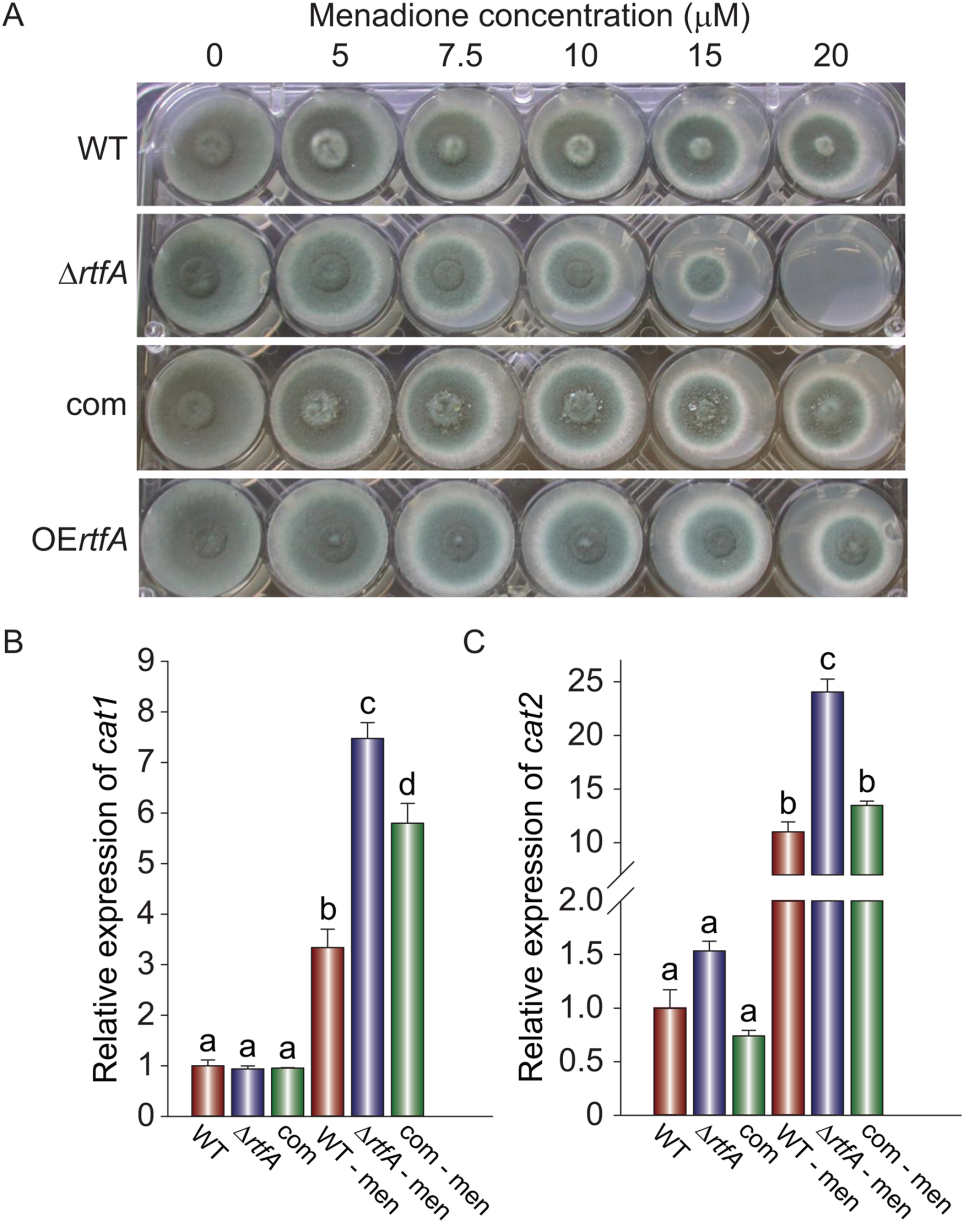
*rtfA* is necessary for normal resistance to oxidative stress. (A) Sensitivity of *A*. *fumigatus* wild type (WT), Δ*rtfA*, complementation (com), and overexpression *rtfA* (OE*rtfA*) strains to oxidative stress was examined on GMM containing various concentrations of menadione as indicated. Colony formation was observed after 72 h of incubation at 37°C. (B, C) In a separate experiment, the strains were inoculated in liquid GMM with or without 20 μM of menadione (men). Cultures were incubated at 30°C. Mycelia were collected 6 h after the shift for expression analysis of *cat1* (B) and *cat2* (C). Different letters above the bars indicate significantly different values (p ≤ 0.05). Error bars represent standard error.

Generation of an oxidative stress environment is an important line of host defense against *A*. *fumigatus* infections [48, 49]. Our study showed that deletion of *rtfA* results in greater sensitivity to oxidative stress, being unable to grow in the presence of 20 μM menadione, a condition that allowed near-normal growth of wild type colonies (Fig 5). Overexpression of *rtfA* did not change the sensitivity to menadione compared to that in the wild type (Fig 5A and S5 Fig). In a separate experiment, the strains were also grown in liquid shaking cultures with or without menadione. Under these conditions, the expression of two genes involved in oxidative stress response, *cat1* and *cat2* [49] was unexpectedly higher in the Δ*rtfA* strain compared to the wild type after 6 h of exposure to menadione (Fig 5B and 5C). Absence or overexpression of *rtfA* did not alter the effect of other stress factors such as pH, temperature or osmostress on the growth of *A*. *fumigatus* (S6, S7, and S8 Figs).

### *rtfA* regulates *A*. *fumigatus* secondary metabolism

**Fig 6:**
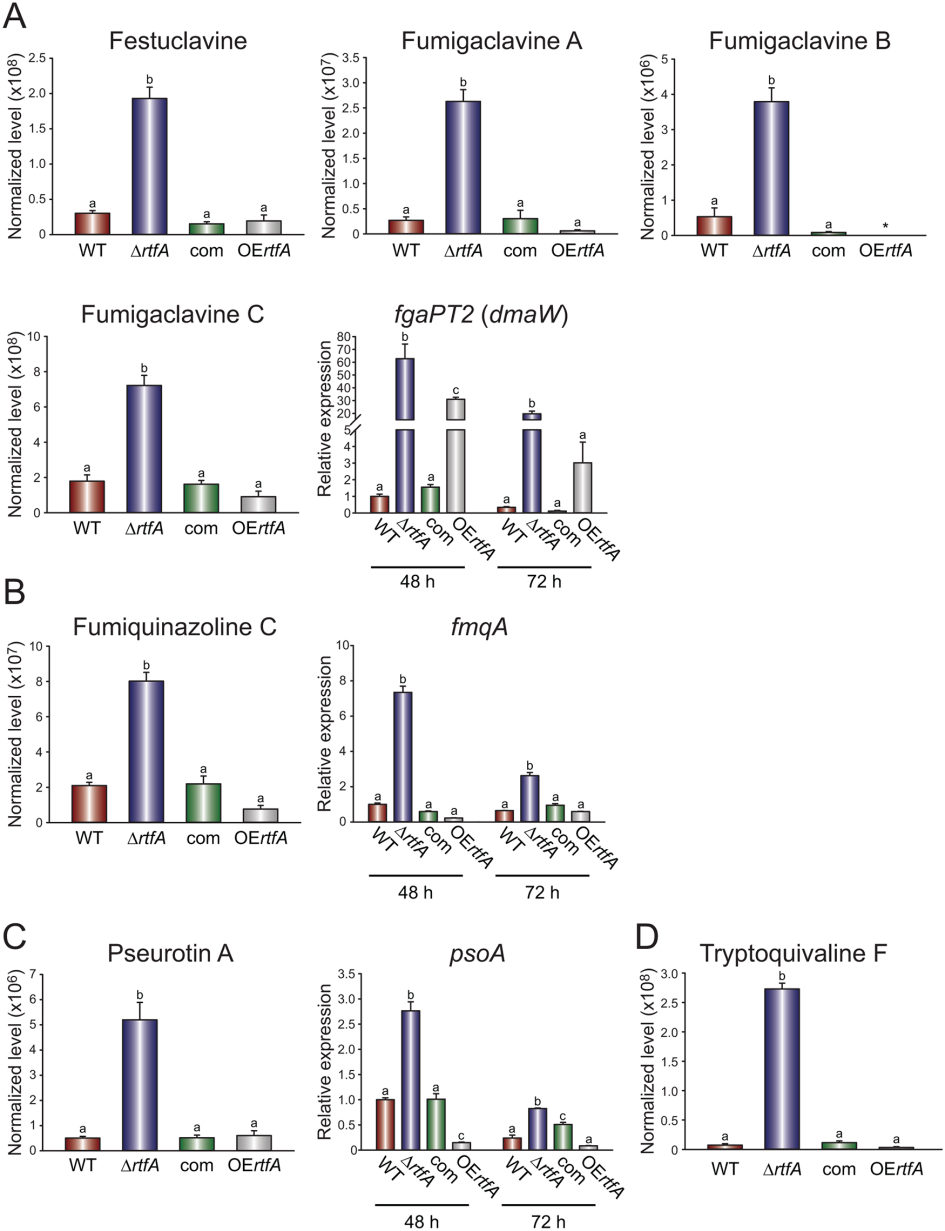
*rtfA* regulates the production of secondary metabolites. Plates containing 25 mL liquid GMM were inoculated with 10^6^ spores/mL of *A*. *fumigatus* wild type (WT), deletion (Δ*rtfA*), complementation (com), and overexpression (OE*rtfA*) strains. Cultures were incubated at 37°C. Mycelia were collected at 48 h and 72 h for expression analysis. Supernatant was collected at 120 h for secondary metabolite extraction and analysis. (A) Analysis of ergot alkaloids and *fgaPT2 (dmaW)*, a key gene in this cluster. (B) Analysis of fumiquinazoline C and *fmqA*, the gene encoding a NRPS involved in fumiquinazoline C biosynthesis. (C) Analysis of pseurotin A and *psoA*, encoding the PKS-NRPS in this biosynthetic pathway. (D) Analysis of tryptoquivaline F production. Different letters above the bars indicate significantly different values (p ≤ 0.05). Error bars represent standard error.

*Aspergillus fumigatus* produces a myriad of secondary metabolites with bioactive properties that serve multiple functions [50], including pathogenicity. We examined whether an assortment of secondary metabolites are *rtfA*-dependent in this fungus. Our LC-MS analysis revealed that various ergot alkaloids, including festuclavine and fumigaclavines A, B, and C, belonging to the same biosynthetic pathway, were produced at significantly higher levels in the *rtfA* mutant strain compared to the wild type (Fig 6A). Overexpression of *rtfA* resulted in undetectable production of fumigaclavine B (Fig 6A). Concomitantly, the gene *fgaPT2*, encoding an enzyme involved in an early step of this pathway [51], was expressed at higher levels in the Δ*rtfA* strain with respect to the controls at both 48 h and 72 h (Fig 6A). Interestingly, expression of *fgaPT2* was also elevated in the overexpression strain compared to wild-type levels, however this increase was not sufficient to increase production of these alkaloids (Fig 6A).

The synthesis of another secondary metabolite, fumiquinazoline C, was also *rtfA*-dependent; production of this compound increased in the absence of *rtfA* (Fig 6B). Expression analysis of the *fmqA* gene, encoding the non-ribosomal peptide synthase (NRPS) in the fumiquinazoline C biosynthetic pathway [52], revealed an increase in transcription in the *rtfA* mutant, while a decrease was observed in the overexpression strains (Fig 6B). Similar results were observed when analyzing levels of pseurotin A production, as well as the expression of the *psoA* gene (Fig 6C), encoding a polyketide synthase-NRPS (PKS-NRPS) involved in the pseurotin A biosynthesis [53]. Furthermore, deletion of *rtfA* also drastically increased the production of tryptoquivaline F (Fig 6D).

### *rtfA* effect on cell wall and adhesion capacity

**Fig 7:**
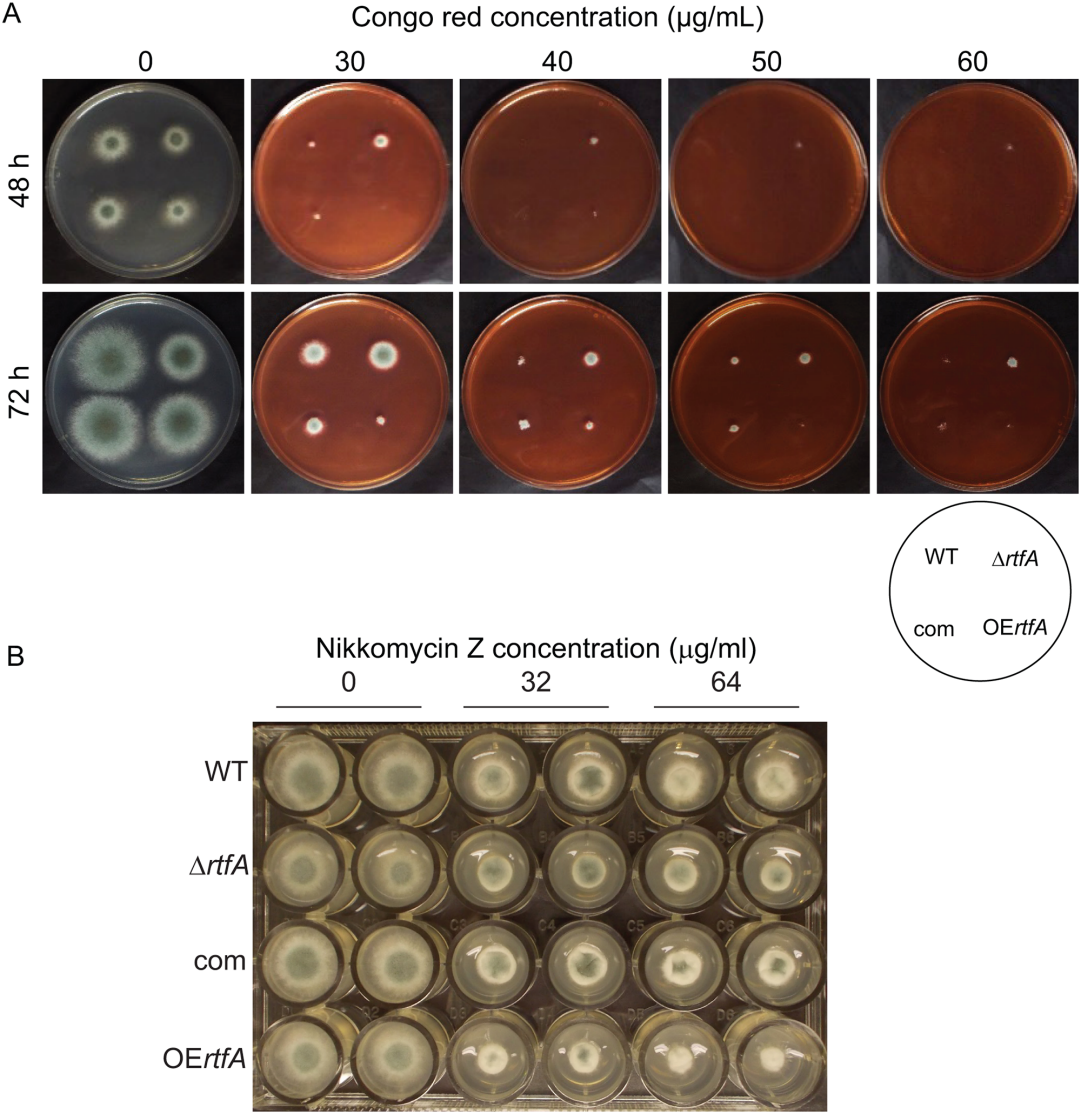
Alteration of *rtfA* expression has a minor effect on cell wall integrity. Wild type (WT), deletion (Δ*rtfA*), complementation (com), and overexpression (OE*rtfA*) strains were point-inoculated on GMM supplemented with increasing concentrations of (A) Congo Red or (B) Nikkomycin Z as indicated. Strains were incubated at 37°C for 72 h (A) or 48 h (B).

*Aspergillus fumigatus* cell wall represents the first point of contact with the host. To investigate the possible role of *A*. *fumigatus rtfA* in cell wall integrity, we subjected Δ*rtfA* and *rtfA* overexpression strains as well as their controls, wild type and complementation strains, to various known cell wall stressors commonly used in *S*. *cerevisiae* and *Aspergillus* studies [54–59]. When grown on medium supplemented with SDS, no noticeable differences were observed (S9 Fig). However, when Congo red or the chitin synthase inhibitor nikkomycin Z [56] were added to the medium, the overexpression strain presented an increase in sensitivity to these agents with respect to the control, while the *rtfA* mutant showed a slight increase in resistance, particularly in the presence of Congo red (Fig 7A and 7B).

In the host biofilm formation has been shown to be important in *A*. *fumigatus* resistance to anti-fungal drugs [60, 61, 62]. Adhesion to surfaces is necessary for the formation of biofilm [63, 63]. Our analysis shows an initial delay in the capacity of this fungus to bind to abiotic surfaces in the absence of *rtfA*. A reduction in adhesion was observed in this mutant at 24 h and 48 h after inoculation (S10 Fig). However, at 72 h, the Δ*rtfA* strain showed wild-type levels of this capacity.

### Deletion of *rtfA* leads to a decrease in virulence in both *Galleria mellonella* and murine infection models

**Fig 8:**
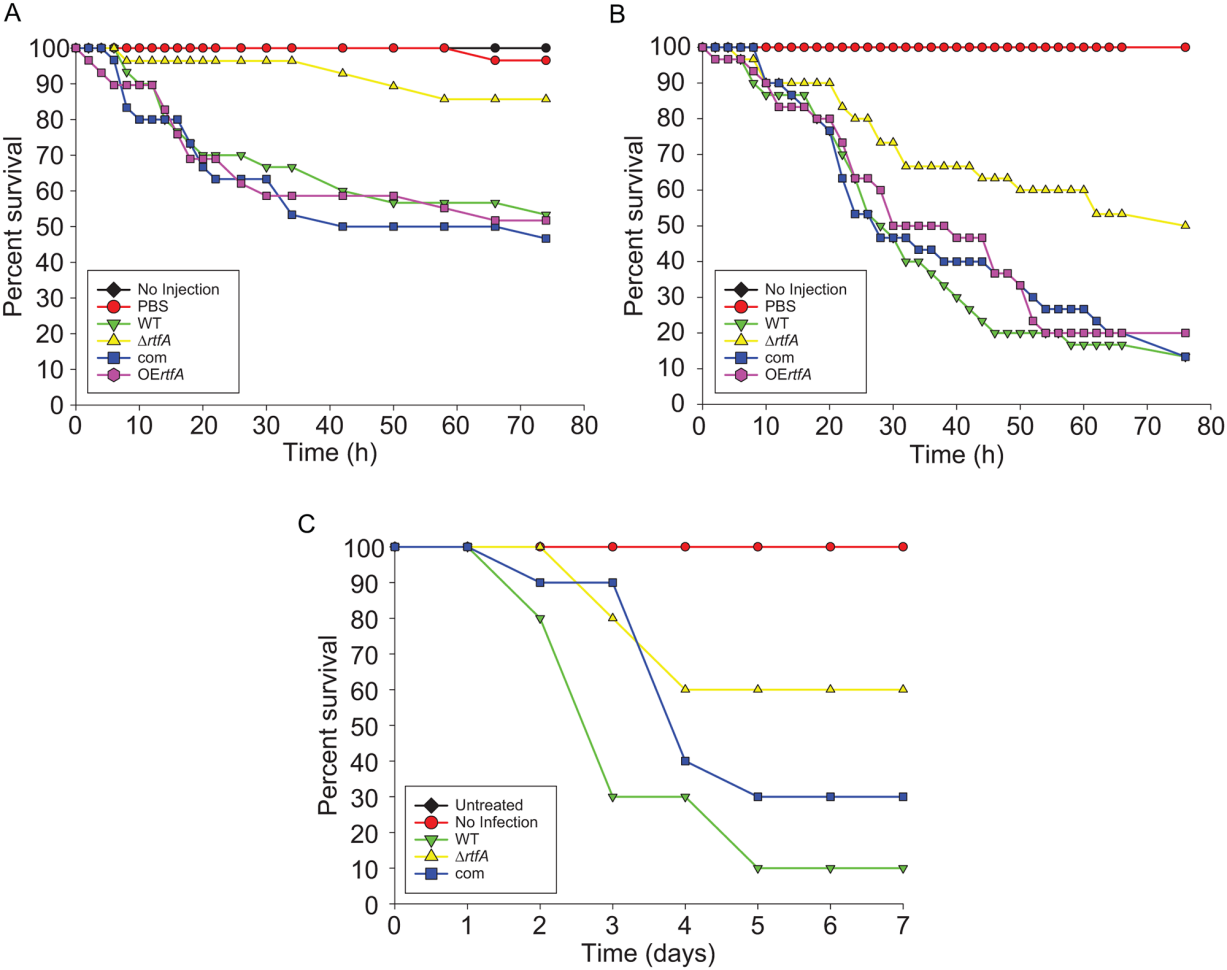
*rtfA* is indispensable for normal pathogenicity. Larvae of *G*. *mellonella* were infected with conidia of *A*. *fumigatus* wild type (WT), Δ*rtfA*, complementation (com), and overexpression *rtfA* (OE*rtfA*) strains as described in the experimental procedures section. Thirty larvae were infected for each group, including a control group injected with 1X PBS. Survival was first monitored every two hours and eventually extended to every 4 or 8 hours when the larval mortality rate decreased. (A) Larvae infected with 10^5^ spores and (B) 10^6^ spores. A no injection control and a PBS control were included. (C) Six-week old mice were rendered neutropenic by treatment with a cyclophosphamide and kenalog-10. Neutropenic mice were infected with 10^6^ conidia per mouse of wild type (WT), Δ*rtfA*, or complementation strains and monitored daily for a total of seven days. Two controls were included, one with a group of non-infected mice rendered neutropenic and a second group with mice neither infected with spores nor treated with immunosuppressants. Statistical analysis was carried out by pairwise comparison using a long rank test.

Since *rtfA* affects growth, conidiation, protease activity, production of secondary metabolites, and response to cell wall and oxidative stresses, it is likely that *rtfA* may play an important role in pathogenicity. To test this possibility *Galleria mellonella* larvae were infected with 10^5^ spores/larva of wild type, Δ*rtfA*, complementation, or OE*rtfA* strains. After 74 hours of observations, only 51% of the larvae infected with wild type survived, while 85% of the larvae infected with Δ*rtfA* were still alive (Fig 8A). Larvae infected with complementation and OE*rtfA* strains showed similar survival rates to that of the wild type. In a similar experiment the larvae were also inoculated with an increased inoculum (10^6^ spores/larva). Under these conditions 87% of the larvae infected with the wild type spores died, and those infected with the complementation and overexpression strains had a similar result, while 50% of the larva infected with the deletion mutant died (Fig 8B). The differences between the wild type and deletion mutant were statistically significant for both inoculum concentrations tested.

Considering the results with the *G*. *mellonella* model, pathogenicity tests were then performed in a mammalian model (Fig 8). A sample size of 10 mice was used per strain. Similar to the results of the pathogenicity tests using the *Galleria* infection model, the mice infected with the mutant strain showed an increased survival rate compared to those infected with the wild-type strain; at the seventh day in this experiment, one mouse of the group infected with the *A*. *fumigatus* wild-type strain was still viable, while six mice were still alive in the group of animals infected with the *rtfA* mutant. Two control groups were used in this experiment. One control group was immunosuppressed but not infected with spores, while the other control group was neither immunosuppressed nor infected with spores. Both groups showed 100% survival.

## Discussion

*Aspergillus fumigatus rtfA* encodes a nuclear putative RNA polymerase II transcription elongation factor-like protein that is homologous to *rtf1* in *Saccharomyces cerevisiae*. Rtf1 is a member of a protein complex called Paf1C that includes the proteins Paf1, Cdc73, Ctr9, and Leo1. Originally thought to simply be an alternative for the Mediator protein complex, a coregulator of transcription [64], subsequent research has shown that the Paf1C associates with the RNA polymerase II from the pre-initiation complex to the 3’ end, where RNA is cleaved from the DNA template [22, 65, 66]. Indeed, Rtf1 has been shown to be the primary agent in binding the Paf1C to RNA Polymerase II [25]. The most studied role of the Paf1C is in the regulation of chromatin structure and histone modifications. In *S*. *cerevisiae*, Rtf1 is involved in regulating the mono-ubiquitylation of histone H2B at lysine 123, which is required for the methylation of histones H3K4 and H3K79 [23, 24, 67–71]. Interestingly, complementation of the *A*. *fumigatus rtfA* mutant with the *S*. *cerevisiae rtf1* gene rescued wild-type phenotype, suggesting that both homologs carry out conserved functions. The fact that the 6-AU treatment did not affect the Δ*rtfA* strain in a similar manner as loss of *rtf1* in *S*. *cerevisiae* [38, 40] does not necessarily indicate a different function for *rtfA*, since mutations in transcription elongation factors do not always bestow sensitivity to 6-AU [72]. It is possible that the RNA Polymerase II could rely more heavily on factors that promote elongation in yeast [24]. This *A*. *fumigatus* study, as well as previous studies in other *Aspergillus* [28, 29] and in yeast [i.e. 22, 65–71], revealed a broad regulatory role for *rtfA*, where this transcriptional regulator controls the expression of many genes, including those involved in conidiation. As previously mentioned, the main route of entry for *A*. *fumigatus* into humans is through inhalation of conidia [11]. Indeed, the conidia produced by *A*. *fumigatus* are ubiquitous in the air and it is estimated that humans inhale a few hundred conidia per day [48]. Our results indicate that *rtfA* is involved in the regulation of conidial production. Deletion of *rtfA* resulted in hyperconidiation with concomitant increase in *brlA* and *wetA* expression levels. Conidiation was also observed even in submerged cultures, a condition that prevented conidiation in the wild type. The effect of *rtfA* on conidiation also varies in different *Aspergillus* species. In contrast with *A*. *fumigatus*, in *A*. *nidulans* and *A*. *flavus* the absence of *rtfA* results in a reduction of conidiation [28, 29]. It is possible that evolutionary rewiring could have occurred resulting in the observed differential regulatory output of *rtfA* in morphological development in different *Aspergillus* species.

The conidia that are able to evade the defenses of the lung environment will germinate and grow as hyphae. In our study, the deletion of *rtfA* lead to colonies that were significantly reduced in vegetative growth (37% decrease) compared to the wild type strain, which may affect the ability of the fungus to establish an infection in the host. These results coincide with previous findings from our lab, where *rtfA* in *A*. *nidulans* and *A*. *flavus* was also shown to affect growth [28, 29], although the effect of the deletion of *rtfA* in *A*. *fumigatus* growth was slightly more severe compared to that of *A*. *nidulans and A*. *flavus*.

The human immune system has an array of ways to defend the organism against pathogens such as *A*. *fumigatus*. Macrophages and neutrophils produce reactive oxygen species (ROS) that can be detrimental to the fungal spores and hyphae [48]. For this reason, we tested the oxidative stress response of *A*. *fumigatus* wild type, Δ*rtfA*, complementation, and OE*rtfA* strains to various concentrations of menadione. Menadione produces ROS once it enters the cell, creating an environment that is under oxidative stress [73]. Our results conclusively showed that *rtfA* is important for the response of *A*. *fumigatus* to oxidative stress, where the mutant was unable to grow on solid medium in the presence of a 20 μM concentration of menadione, compared to the wild type strain which was still viable at this concentration. Unexpectedly, when gene expression of *cat1* and *cat2* was assessed in submerged cultures, the Δ*rtfA* strain presented higher expression compared to the wild type, indicating that *rtfA* negatively regulates the expression of these genes. Both *cat1* and *cat2* encode catalases found within the fungal mycelium, in contrast to *catA*, which is only produced in the conidia [74]. Deletion of *cat1* or *cat2* does not attenuate virulence of *A*. *fumigatus in vivo*, however a double deletion mutant did increase survival [49]. Despite this, it is difficult to assess the inclusion of catalases as virulence factors due to redundancy; the fungus possesses many genes that aid in combating ROS, including five catalases (*catA*, *cat1*/*catB*, *catC*, *catE*, and *cat2*/*katG*) and four superoxide dismutatases: sod1, sod2, sod3, and sod4 [49, 74, 75]. It is possible that although *cat1* and *cat2* are expressed at higher levels in the mutant strain, *rtfA* could be required for other factors involved in oxidative stress resistance.

Although *A*. *fumigatus* is known as an important opportunistic human pathogen, this fungus predominantly lives as a saprophytic organism, being found on dead and decaying organic matter. In such an environment, it is important for this organism to produce a plethora of hydrolases in order to obtain nutrients [76]. A majority of these degradative enzymes are specific for plant cell wall components; nevertheless, this fungus does possess some enzymes that can target human tissue, including proteases such as elastase [48, 77]. This is important during lung infection, a microenvironment rich in elastin. In our study we found that the *rtfA* mutant strain presented a reduction in protease activity with respect to the wild type, indicating the importance of *rtfA* on nutrient acquisition, and possibly contributing to *A*. *fumigatus* pathogenicity.

*Aspergillus fumigatus* is also known to produce a number of secondary metabolites with a vast spectrum of biological activities. These include a family of ergot alkaloids and the peptidyl alkaloids fumiquinazoline C and tryptoquivaline F, which have been experimentally shown to be associated with conidia and thus dependent on *brlA* [35, 52, 78, 79]. Though the effect of overexpressing *brlA* was not examined in that case, it is interesting to note that in our studies the *rtfA* deletion mutant presented an increase in *brlA* expression and conidia along with an increase in the production of the ergot alkaloids festuclavine and fumigaclavines A, B, and C, as well as the increase in the peptidyl alkaloid fumiquinazoline C and tryptoquivaline F production. The significance of these classes of alkaloids is revealed in the possible therapeutic applications due to their cytotoxic, anti-inflammatory, anti-tumor, and anti-cancer activities [80, 81]. Another secondary metabolite produced by *Aspergillus sp*., pseurotin A, has also demonstrated anti-tumor activity as well as anti-parastic activity [82]. Our current study shows that *rtfA* is a positive regulator of pseurotin A production in *A*. *fumigatus*.

The absence of *rtfA* did not lead to major effects on the integrity of the cell wall, as suggested by the presence of fungal growth when the *rtfA* mutant was exposed to chemical agents such as SDS. However, our studies indicate that this gene is necessary for normal adhesion capacity to surfaces. This adhesiveness is necessary for the formation of biofilm [83], which also forms *in vivo* when the fungus is infecting humans [61]. The significance of biofilm is seen in patients infected with a fungal pathogen, where it has been demonstrated in both *A*. *fumigatus* as well as *Candida albicans* to decrease antifungal drug susceptibility [60, 84, 85].

The fact that absence of *rtfA* results in pleiotropic effects such as alterations in vegetative growth and morphogenesis, resistance to oxidative stress, protease activity, as well as proper adhesion capacity in *A*. *fumigatus* could contribute to variations in virulence in this mutant. Indeed, our *in vivo* analysis of the effect of *rtfA* on pathogenicity in the larvae of the greater wax moth, *Galleria mellonella*, revealed a role of *rtfA* in virulence, as the larvae infected with the deletion mutant showed a significant increase in survival rate compared with those infected with *A*. *fumigatus* wild type. *Galleria mellonella* is often used as a preliminary model system to test virulence due to its cost effectiveness, feasibility, and possession of an innate immune system [86–88]. With these results in mind, we tested an additional *in vivo* model using neutropenic murine animals. In this second model, the Δ*rtfA* strain also showed a reduction in virulence compared to that of the wild type, indicating that *rtfA* is indispensable for establishing normal infections.

In conclusion, this study demonstrates the involvement and importance of the *rtfA* gene in *A*. *fumigatus*. Fundamental biological processes, such as growth and morphological development are regulated by *rtfA*. We also demonstrated the influence of *rtfA* on the synthesis of various secondary metabolites as well as protease activity, an important element in nutrient acquisition that could also influence pathogenicity. Other factors also associated with virulence, such as oxidative stress and adhesion capacity were affected by *rtfA* as well. The profound effect of *rtfA* in *A*. *fumigatus* biology makes this gene indispensable for normal pathogenicity. Indeed, possibly the most relevant finding was the significant decrease in virulence in the absence of *rtfA*, shown in both the *Galleria* and murine infection models. This reduction in virulence, together with the fact that *A*. *fumigatus* RtfA presents low similarity with homologs of higher eukaryotes, suggest that this regulator could be used as a potential desirable target in the design of a control strategy against Aspergillosis.

## Supporting information

S1 Table Primers used in this study. (DOCX)

S2 Table Strains used in this study. (DOCX)

S1 Fig Generation of the *A*. *fumigatus rtfA* deletion, complementation and over-expression strains. (A) Schematic diagram showing the replacement of *rtfA* with the *A*. *parasiticus pyrG* gene by a double-crossover event. Southern blot analysis confirming the proper integration of the cassette is shown. The 5’ UTR was used as the probe. SalI (S) was used to digest genomic DNA. Bands of 5.1 kb and 3.7 kb indicate deletion of *rtfA* in the mutant (Δ*rtfA*), whereas bands of 5.1 and 1.6 correspond to the wild type (WT, CEA10). The deletion strain was denominated TRRM4. (B) Confirmation of the complementation strain by PCR using primers AfumRM3_Oef (F) and AfumRM3_Oer (R) (S1 Table). The expected PCR product size in the complementation strain is 2036 bp. Wild type (CEA10) genomic DNA was used as a positive control and Δ*rtfA* DNA was used as negative control. (C) Generation of the over-expression strain was confirmed by PCR analysis, using primers gpdApromoF (F) and AfumRM3_oer (R). The expected product size is 2178 bp. The plasmid used for transformation (pRRM2) was used as positive control. (D) qRT-PCR expression analysis of *rtfA* in the four strains obtained. The results were normalized to the WT considered as 1. Bars represent standard error. Asterisk: not detected.
(PDF)

S2 Fig Assessment of the effect of 6-azauracil on *A*. *fumigatus* growth in the presence, absence or overexpression of *rtfA*. Wild type (WT), deletion (Δ*rtfA*), complementation (com), and overexpression (OE*rtfA*) strains were point-inoculated on GMM supplemented with increasing concentrations of 6-Azauracil. Strains were incubated at 37°C. (A) Photos correspond to 48 h and 72 h cultures (upper and lower rows respectively). Colony diameters were measured at 48 h (B) and 72 h (C). Error bars represent standard error.
(PDF)

S3 Fig *rtfA* affects conidiation and pigmentation. (A) Quantification of conidial production on 1% GMM agar plates. Strains were incubated at 37°C for six days and core samples were obtained 0.5 cm from the colony center. Different letters above the bars indicate significantly different values (p ≤ 0.05). Error bars represent standard error. (B) Cultures of Δ*rtfA* grown in submerged cultures (GMM) accumulated a purple pigment not present in the other strains.
(PDF)

S4 Fig *rtfA* localizes in nuclei. (A) Diagram of the strategy to fuse *gfp* to *rtfA*. The transformation cassette contained 885 bp of the *rtfA* 3’ region. The tagged construct was introduced at the *rtfA* locus by a double-recombination event as indicated, resulting in the TRRM6 strain. Transformants were verified by diagnostic PCR with primers AfumRM3_Oef (F) and AfumRM33Nested (R) (S1 Table). The expected PCR products for the *gfp*-tagged strain (5.9 kb) and wild type (3.2 kb) were obtained (right) (B) Micrographs showing the subcellular localization of RtfA::GFP. From left to right, DIC images, DAPI images indicating the position of nuclei, and green fluorescence (GF) images. Arrows indicate nuclei. Scale bars represent 10 μm.
(PDF)

S5 Fig Evaluation of the effect of *rtfA* overexpression on oxidative stress sensitivity. The wild type (WT) and overexpression (OE*rtfA*) strains were tested in the presence of a range of menadione concentrations from 0 to 35 μM. Strains were incubated at 37°C. Photograph was taken after 48 h.
(PDF)

S6 Fig Assessment of the effect of pH on *A*. *fumigatus* growth in the presence, absence or overexpression of *rtfA*. *Aspergillus fumigatus* wild type (WT), Δ*rtfA*, complementation (com), and overexpression *rtfA* (OE*rtfA*) strains were point-inoculated on GMM with different pH values and incubated at 37°C. Colony diameters were measured at 48 h (A) and 72 h (B). Images of the plates were taken at 72 h (C).
(PDF)

S7 Fig The effect of temperature on *A*. *fumigatus* growth is not *rtfA*-dependent. *Aspergillus fumigatus* wild type (WT), Δ*rtfA*, complementation (com), and overexpression *rtfA* (OE*rtfA*) strains were point-inoculated on GMM and incubated at a range of temperatures as shown. Colony diameter (A) and images of the plates (B) were obtained at 48 h.
(PDF)

S8 Fig Assessment of the effect of osmotic stress on *A*. *fumigatus* growth in the presence, absence or overexpression of *rtfA*. Wild type (WT), deletion (Δ*rtfA*), complementation (com), and overexpression (OE*rtfA*) strains were point-inoculated onto GMM medium supplemented with 1.2 M sorbitol, 0.6 M KCl, or 1.0 M sucrose to induce osmotic stress. Plates were incubated at 37°C and colony diameters were measured at 48 h (A). Error bars represent standard error. (B) Photographs of colonies from 48 h of incubation.
(PDF)

S9 Fig Effect of SDS on the growth of *A*. *fumigatus* strains with an altered *rtfA* locus. Wild type (WT), deletion (Δ*rtfA*), complementation (com), and overexpression (OE*rtfA*) strains were point-inoculated on GMM supplemented with increasing concentrations of SDS. Strains were incubated at 37°C. (A) Photographs of colony at 48 h and 72 h. (B) Colony diameters measured at 48 h and 72 h after inoculation. Error bars represent standard error.
(PDF)

S10 Fig The role of *rtfA* in adherence to abiotic surfaces. Strains were inoculated in liquid GMM and incubated at 37°C for 24 h (A), 48 h (B), and 72 h (C). After staining with Crystal Violet the absorbance was read at 560 nm. Samples were diluted 3 fold. Different letters indicate statistically different values (p ≤ 0.05). Error bars represent standard error.
(PDF)
