# Supplementary material for: rtfA controls development, secondary metabolism, and virulence in Aspergillus fumigatus
Source: PLoS One. 2017 Apr 28;12(4):e0176702. doi: 10.1371/journal.pone.0176702 (PMC5409149; doi:10.1371/journal.pone.0176702)
Supplement: S1 Table — (DOCX) [file pone.0176702.s001.docx]

**Table S1. Primers used in this study**

| Primer name | Sequence (5'🡺3') |
| --- | --- |
| gdpApromoF | AAGTACTTTGCTACATCCATACTCC |
| AfumRM3_5f | CCTGATCTGAGGATGGCATACTGC |
| AfumRM3_5r | TCAGCGACAGTTTTGCAACGATATTCACT |
| AfumRM3_3f | TTGTGACATCATCTAATCATCGTTGCATAA |
| AfumRM3_3r | CGCAGGTCATACTGAGGAGAACTCG |
| AparapyrGg_fumRM3_f | TCAGCGACAGTTTTGCAACGATATTCACTGGATCCTATGGATCTCAGAACAATATACC |
| AparapyrGg_fumRM3_r | TAGTTTGGGTAAGGGTGATGTCGACTTGTGACATCATCTAATCATCGTTGCATAA |
| AfumRM3_Oef | AAAAGGCGCGCCATGGATGGCGGACGATCTGGATGC |
| AfumRM3_Oer | AAAAAAAAAAAGCGGCCGCTCAGATTTCAATATCCAACTCCAGGTCC |
| afumrm3f | AAAGCCCGAACGGTCGGTCG |
| afumrm3r | GATTCCATTATCCAACTCCAGGTCCAGG |
| afumrm3gfpr | CGCATCAGTGCCTCCTCTCAGACTTGTGACATCATCTAATCATCGTTGCATAA |
| AfumRM35_Nested | GGCCATACAAGTGGTGGCTAGCAGTCTCTC |
| AfumRM33_Nested | CCCGTGTTCGCGCGCTCATTATGC |
| AfRM3_compFNot1 | GCGAAAGTCTGGAGGTGCCAAGGG |
| AfRM3_compRAsc1 | GCCCGAACGAAGAGCTCTGACCAA |
| AfumCatBF | GTCACGGTGGCCCCAACTTT |
| rm3exon2Afum (F) | CGATTCTGAGGGAGCAAGTGAGGACGCTGAAGAAGA |
| rm3exon2Afum (R) | CGCGAGATGCTAAGAGACGGCGAAGAGCCAAGTC |
| afumrm3gfpf | CCTGGACCTGGAGTTGGATATTGAAATCGGAGCTGGTGCAGGCGCTGGAGCC |
| AfumCatBqrtPCR R | GTCTTGGGCGAGTACGCGTGA |
| Afum_dmaW_F | GCCAATGCCTCCAGTGCG |
| Afum_dmaW_R | CGAGACTGGGAATTACGCACTCCT |
| Afum_fqF_F | CCTTCACTTTCGGGGTGTCTCTGA |
| Afum_fqF_R | CGCAGATTTGGAACAGCCTCGG |
| Afum_psoA_F | CTCTGGCGGCGAGATTGGTT |
| Afum_psoA_R | CCGCCCTTCTTTCCATCCTTCC |
| Scer_rtf1_Afum_rtfA_f | TCAGCGACAGTTTTGCAACGATATTCACTATGTCTGATTTAGATGAGGATTTATTAGCCTTG |
| Scer_rtf1_Afum_rtfA_r | GCGCTCATCATGCTCTTCCAGCCTAAAACTTAAGGTCAAATTTGATATCCAATTCACC |
| Afum_rtfA_3f | GCTGGAAGAGCATGATGAGCG |
| Afum_cat2_f | GCAGTCGTCGCAGCCCA |
| Afum_cat2_r | CCAGCGGGTTGGAGACGG |
| Afum_rtfA_nested_f | AAAAAAAAAAAGCGGCCGCCACAATTGGGCGAAGGAGATGG |
| Afum_rtfA_nested_r | AAAAAAAAAAAGCGGCCGCGGCCGCTGCTGTGTTAGC |
| Scer_rtf1_qPCR_F | AGAGCGATCCTTTCAGCAGGCT |
| Scer_rtf1_qPCR_R | GCTCCAACCCACCAAGGC |
