# Supplementary material for: rtfA controls development, secondary metabolism, and virulence in Aspergillus fumigatus
Source: PLoS One. 2017 Apr 28;12(4):e0176702. doi: 10.1371/journal.pone.0176702 (PMC5409149; doi:10.1371/journal.pone.0176702)
Supplement: S2 Table — (DOCX) [file pone.0176702.s002.docx]

**Table S2. Strains used in this study**

| Name | Genotype | Source |
| --- | --- | --- |
| CEA10 | Wild type | Gift from Robert Cramer |
| CEA17 | *pyrG1* | Gift from Robert Cramer |
| KU80Δ | *pyrG1*, Δ*akuB^KU80^* | Gift from Robert Cramer |
| TRRM2 | *pyrG1*, *gpdA*(p)::*rtfA*::*trpC*(t)::*pyrG^A. fum^* | This study |
| TRRM3 | *pyrG1*, Δ*akuB^KU80^*, Δ*rtfA*::*pyrG^A. para^* | This study |
| TRRM4 | *pyrG1*, Δ*rtfA*::*pyrG^A. para^* | This study |
| TRRM5 | *pyrG1*, Δ*rtfA*::*pyrG^A. para^*, *rtfA::ptrA* | This study |
| TRRM6 | *pyrG1*, *rtfA::gfp::pyrG^A. fum^* | This study |
| TRRM7 | *pyrG1*, Δ*akuB^KU80^*, Δ*rtfA*::*pyrG^A. para^*, *rtf1::ptrA* | This study |
