# Supplementary figures and images for: rtfA controls development, secondary metabolism, and virulence in Aspergillus fumigatus
Source: PLoS One. 2017 Apr 28;12(4):e0176702. doi: 10.1371/journal.pone.0176702 (PMC5409149; doi:10.1371/journal.pone.0176702)

A

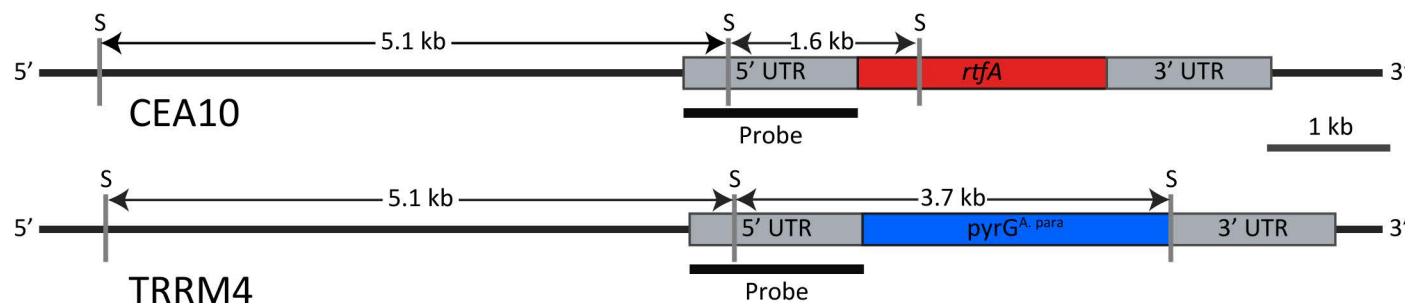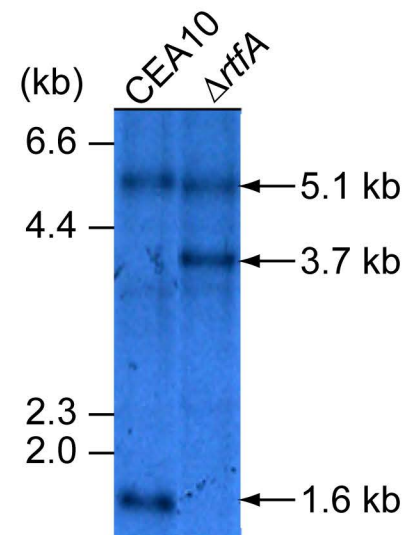

B

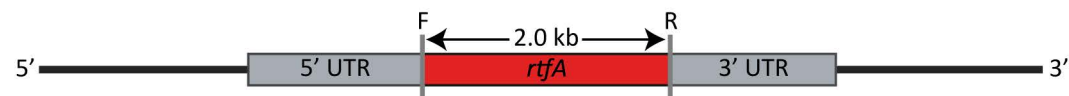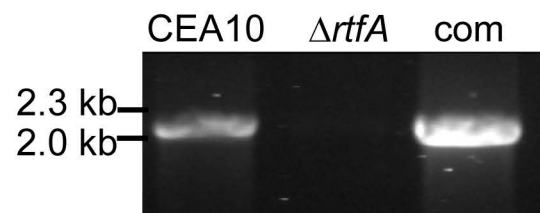

C

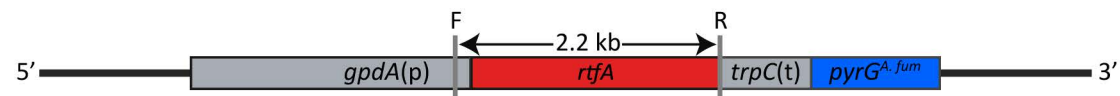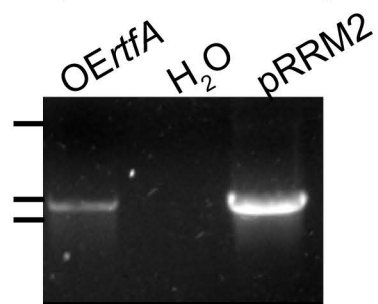

D

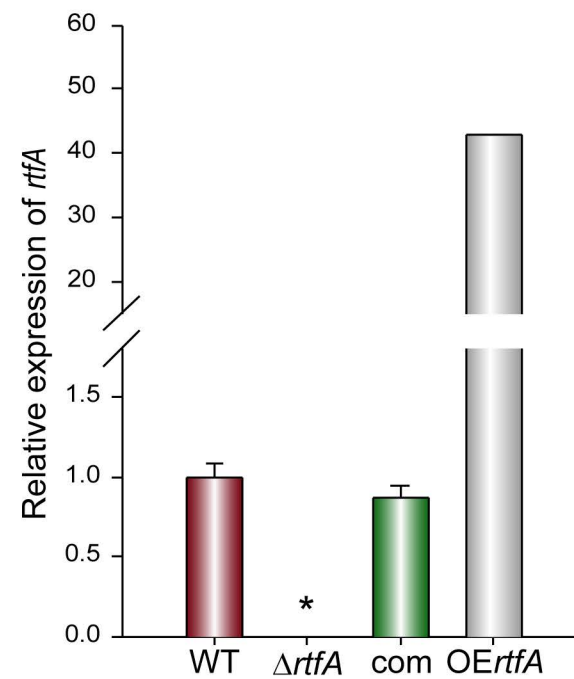

Supplement: S1 Fig — (A) Schematic diagram showing the replacement of rtfA with the A. parasiticus pyrG gene by a double-crossover event. Southern blot analysis confirming the proper integration of the cassette is shown. The 5’ UTR was used as the probe. SalI (S) was used to digest genomic DNA. Bands of 5.1 kb and 3.7 kb indicate deletion of rtfA in the mutant (ΔrtfA), whereas bands of 5.1 and 1.6 correspond to the wild type (WT, CEA10). The deletion strain was denominated TRRM4. (B) Confirmation of the complementation strain by PCR using primers AfumRM3_Oef (F) and AfumRM3_Oer (R) (S1 Table). The expected PCR product size in the complementation strain is 2036 bp. Wild type (CEA10) genomic DNA was used as a positive control and ΔrtfA DNA was used as negative control. (C) Generation of the over-expression strain was confirmed by PCR analysis, using primers gpdApromoF (F) and AfumRM3_oer (R). The expected product size is 2178 bp. The plasmid used for transformation (pRRM2) was used as positive control. (D) qRT-PCR expression analysis of rtfA in the four strains obtained. The results were normalized to the WT considered as 1. Bars represent standard error. Asterisk: not detected. (PDF) [file pone.0176702.s003.pdf]

A

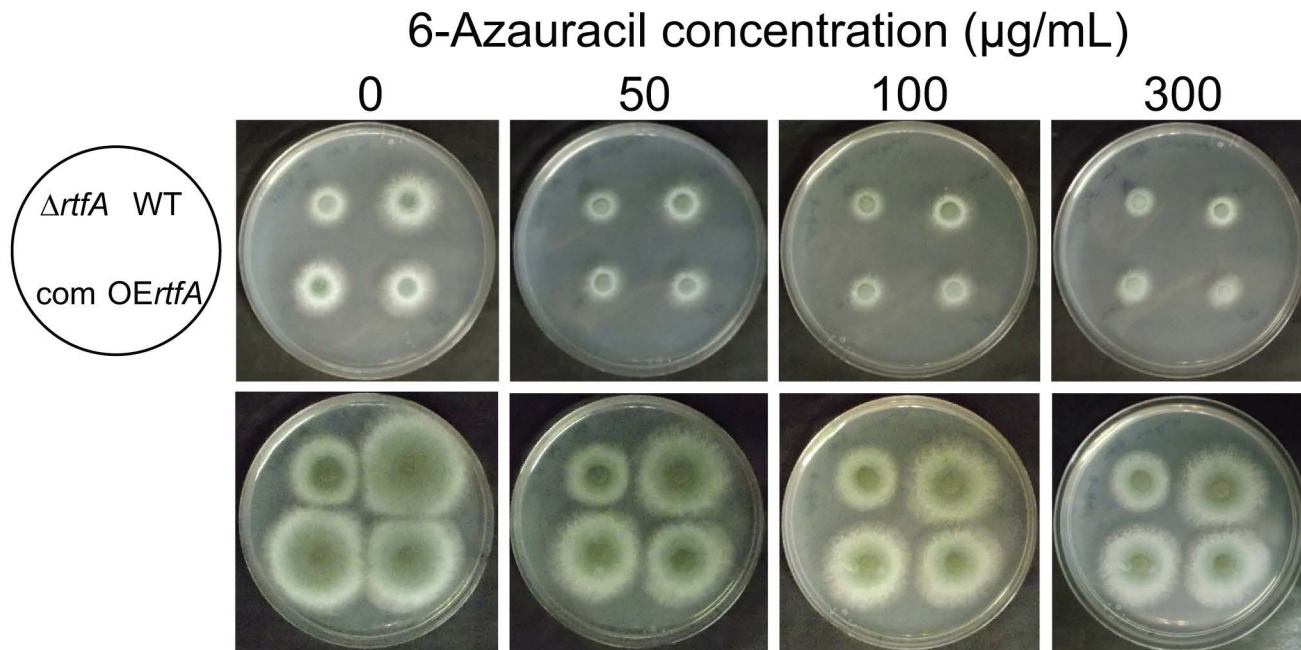

B

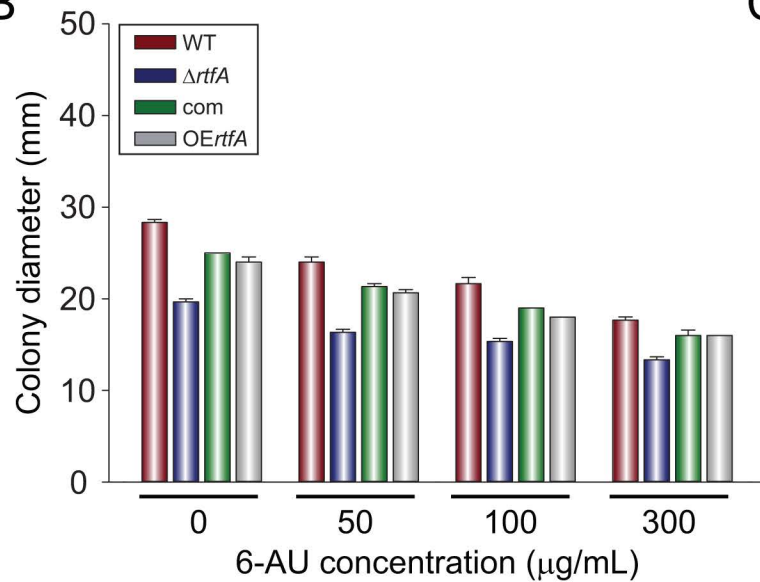

C

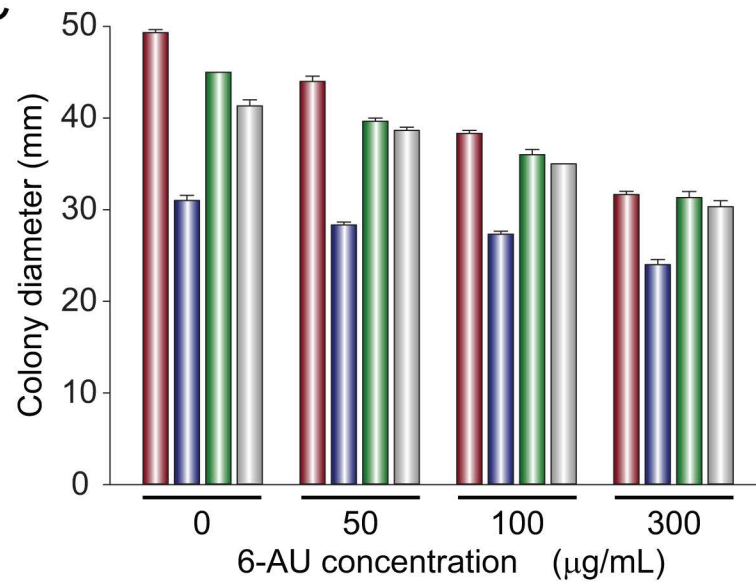

Supplement: S2 Fig — Wild type (WT), deletion (ΔrtfA), complementation (com), and overexpression (OErtfA) strains were point-inoculated on GMM supplemented with increasing concentrations of 6-Azauracil. Strains were incubated at 37°C. (A) Photos correspond to 48 h and 72 h cultures (upper and lower rows respectively). Colony diameters were measured at 48 h (B) and 72 h (C). Error bars represent standard error. (PDF) [file pone.0176702.s004.pdf]

A

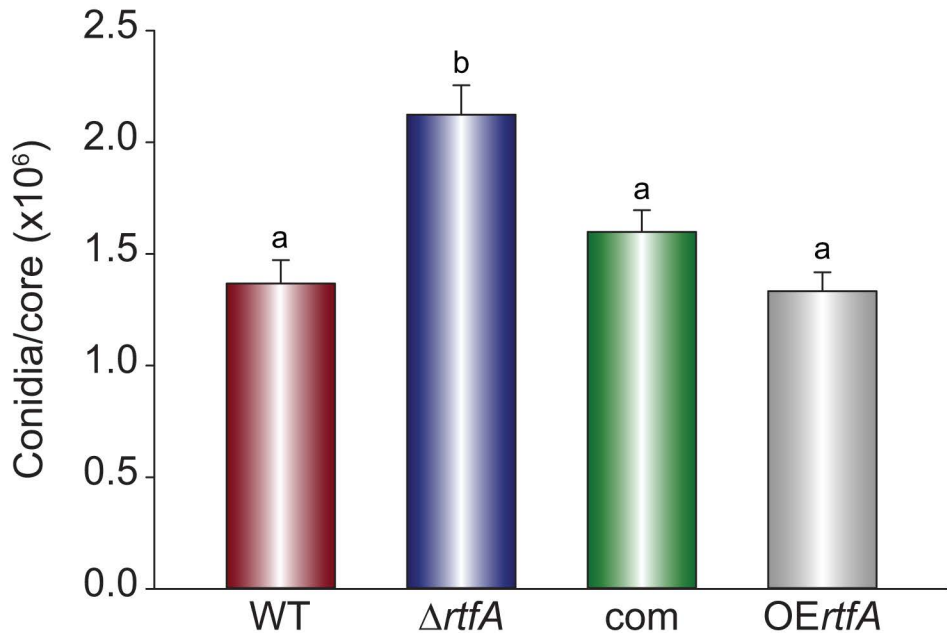

B

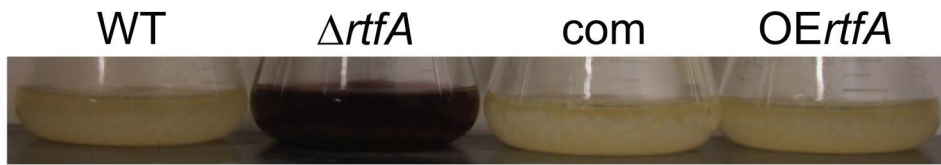

Supplement: S3 Fig — (A) Quantification of conidial production on 1% GMM agar plates. Strains were incubated at 37°C for six days and core samples were obtained 0.5 cm from the colony center. Different letters above the bars indicate significantly different values (p ≤ 0.05). Error bars represent standard error. (B) Cultures of ΔrtfA grown in submerged cultures (GMM) accumulated a purple pigment not present in the other strains. (PDF) [file pone.0176702.s005.pdf]

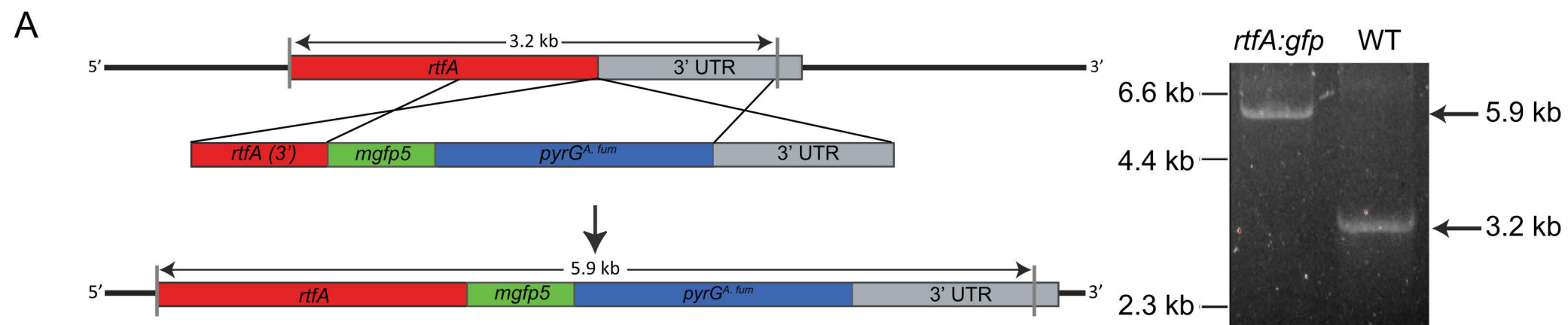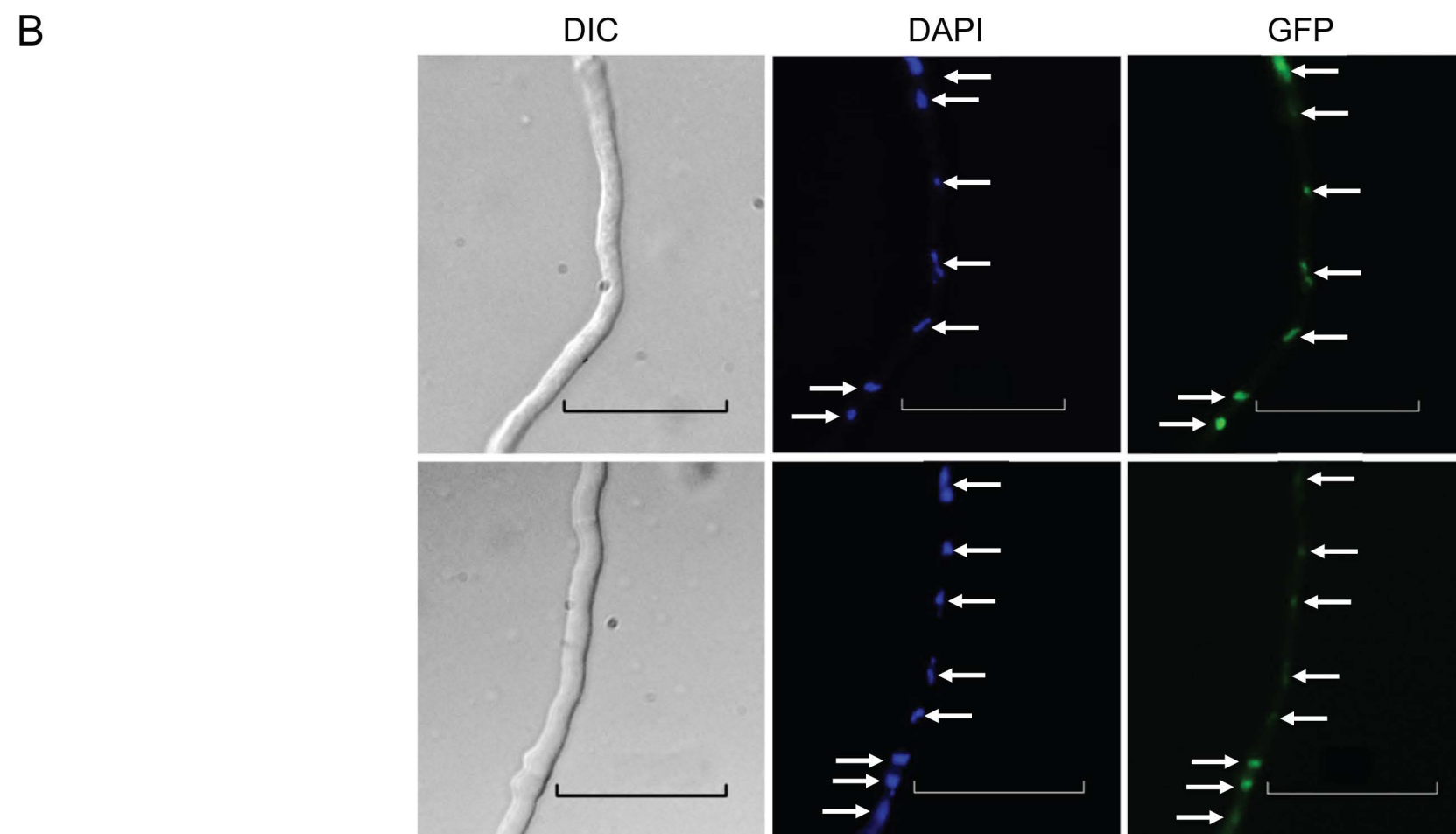

Supplement: S4 Fig — (A) Diagram of the strategy to fuse gfp to rtfA. The transformation cassette contained 885 bp of the rtfA 3’ region. The tagged construct was introduced at the rtfA locus by a double-recombination event as indicated, resulting in the TRRM6 strain. Transformants were verified by diagnostic PCR with primers AfumRM3_Oef (F) and AfumRM33Nested (R) (S1 Table). The expected PCR products for the gfp-tagged strain (5.9 kb) and wild type (3.2 kb) were obtained (right) (B) Micrographs showing the subcellular localization of RtfA::GFP. From left to right, DIC images, DAPI images indicating the position of nuclei, and green fluorescence (GF) images. Arrows indicate nuclei. Scale bars represent 10 μm. (PDF) [file pone.0176702.s006.pdf]

Menadione ( $\mu\text{M}$ )

0

10

20

25

30

35

WT

OErtfA

WT

OErtfA

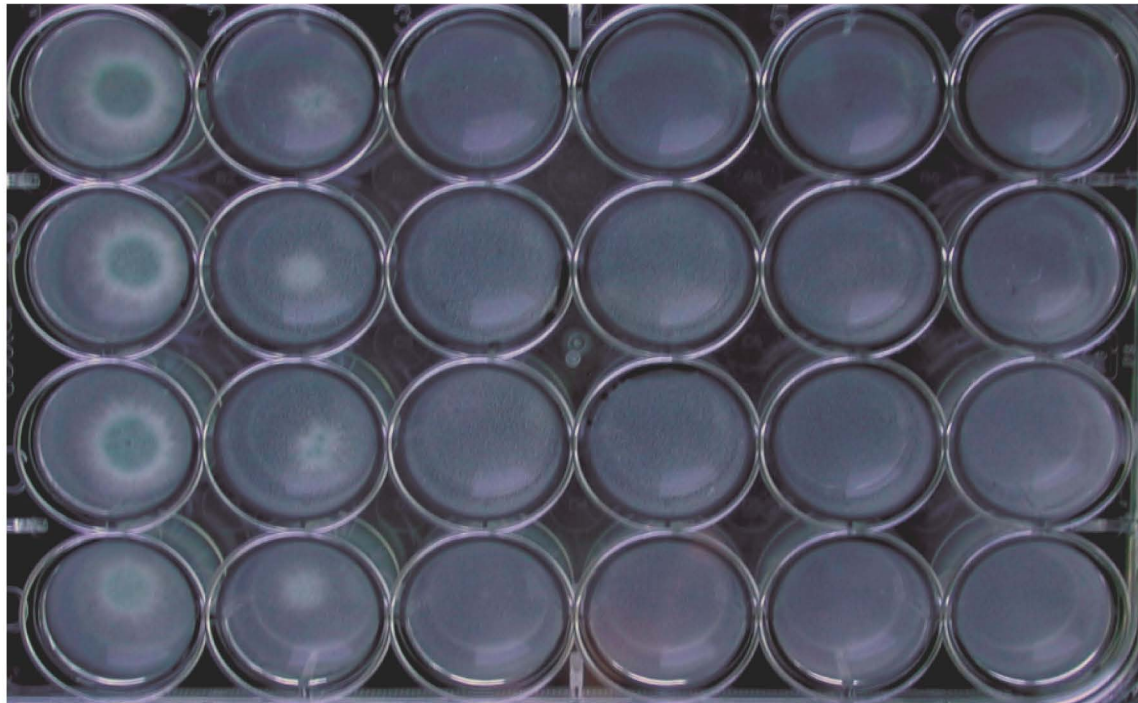

Supplement: S5 Fig — The wild type (WT) and overexpression (OErtfA) strains were tested in the presence of a range of menadione concentrations from 0 to 35 μM. Strains were incubated at 37°C. Photograph was taken after 48 h. (PDF) [file pone.0176702.s007.pdf]

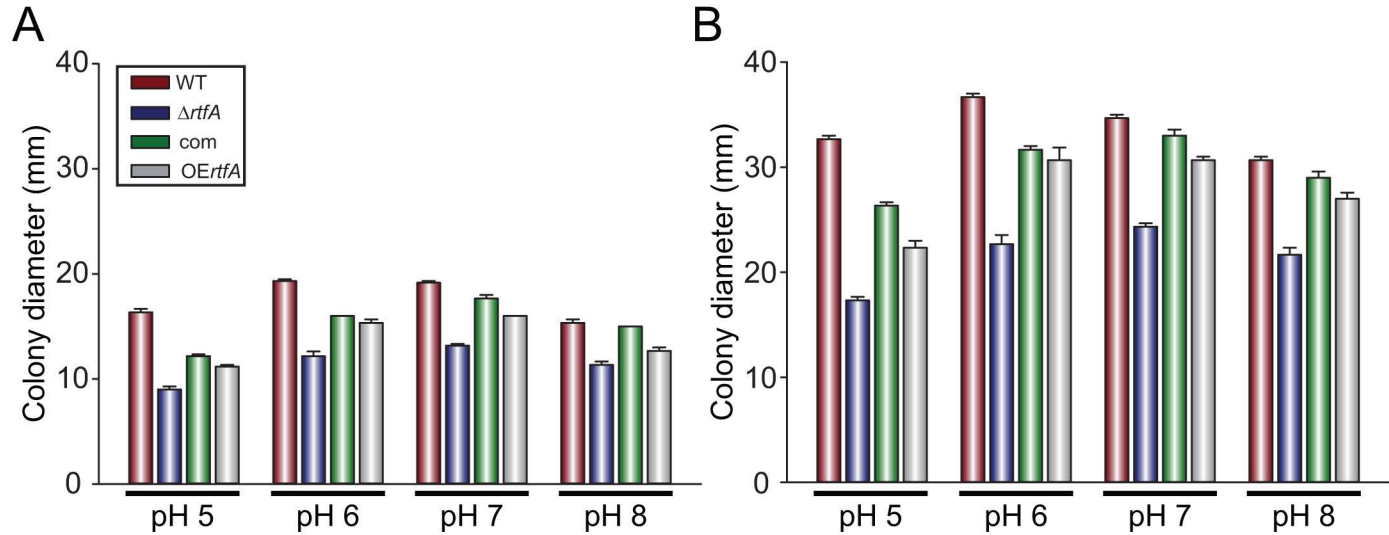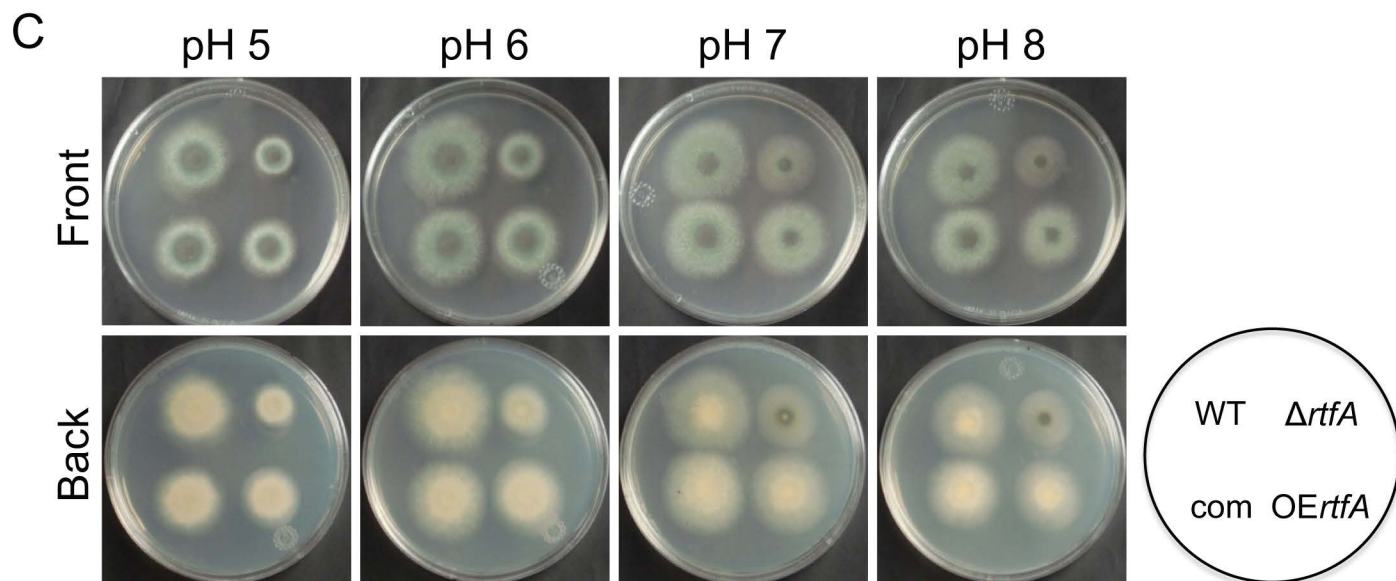

Supplement: S6 Fig — Aspergillus fumigatus wild type (WT), ΔrtfA, complementation (com), and overexpression rtfA (OErtfA) strains were point-inoculated on GMM with different pH values and incubated at 37°C. Colony diameters were measured at 48 h (A) and 72 h (B). Images of the plates were taken at 72 h (C). (PDF) [file pone.0176702.s008.pdf]

A

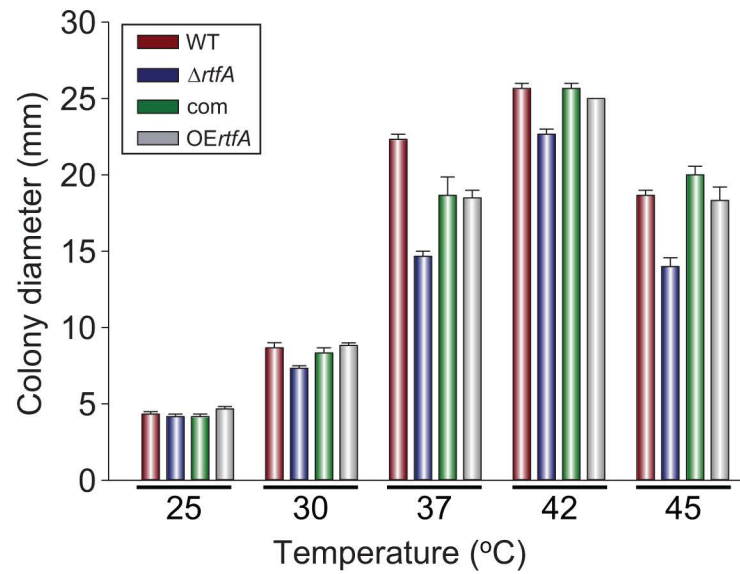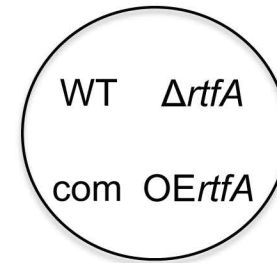

B

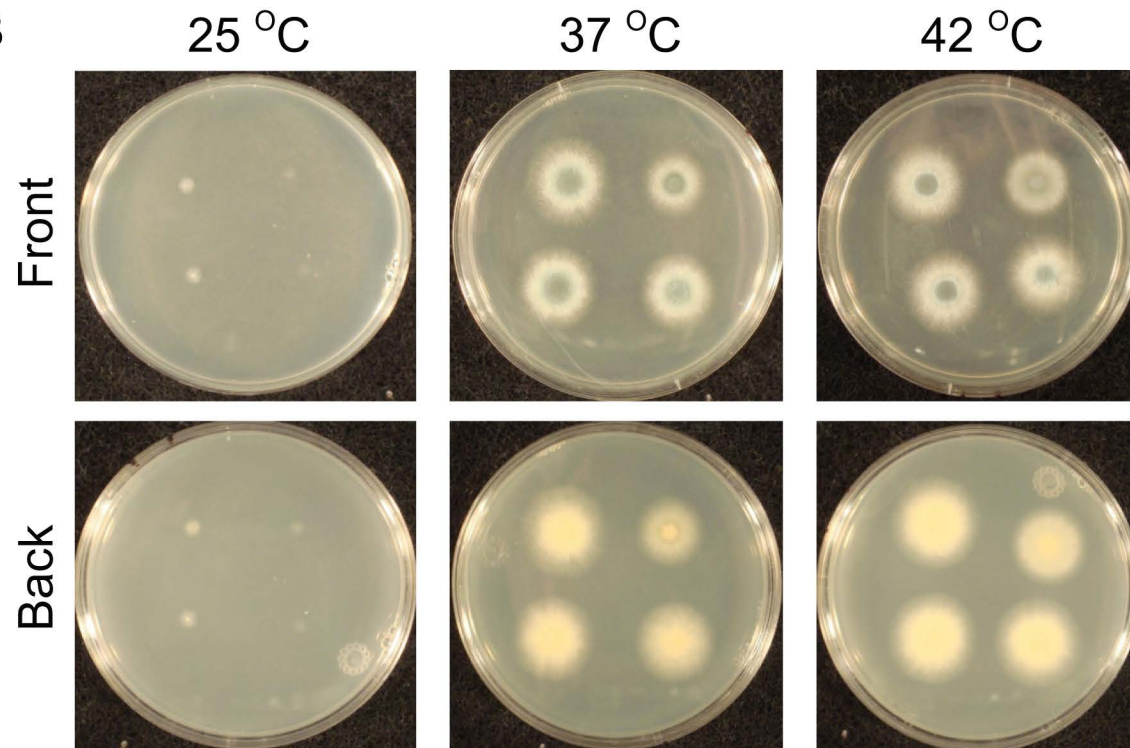

Supplement: S7 Fig — Aspergillus fumigatus wild type (WT), ΔrtfA, complementation (com), and overexpression rtfA (OErtfA) strains were point-inoculated on GMM and incubated at a range of temperatures as shown. Colony diameter (A) and images of the plates (B) were obtained at 48 h. (PDF) [file pone.0176702.s009.pdf]

A

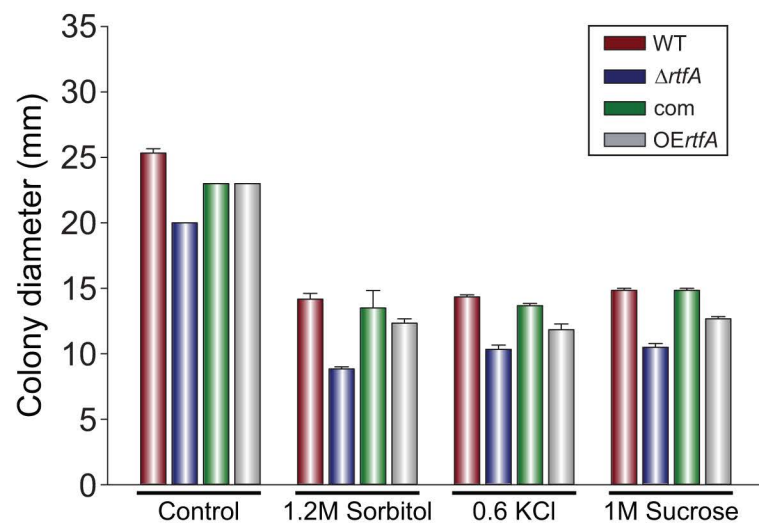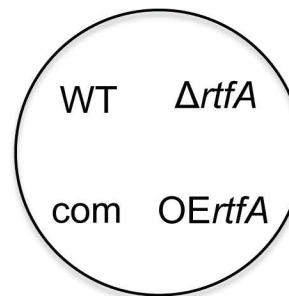

B

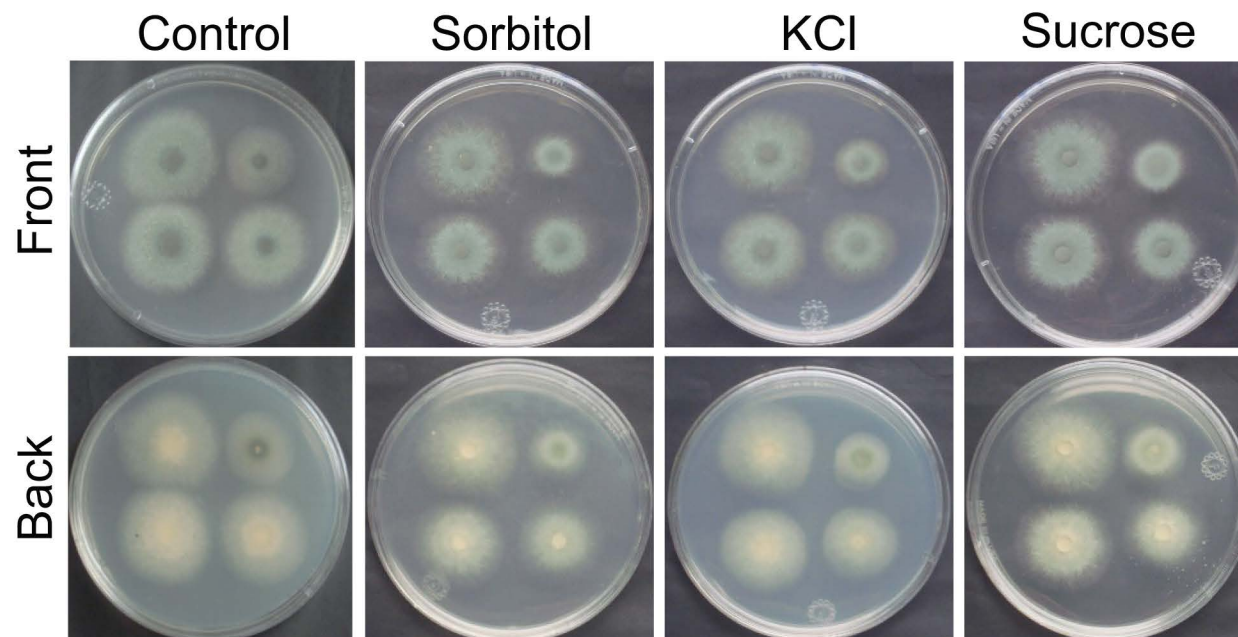

Supplement: S8 Fig — Wild type (WT), deletion (ΔrtfA), complementation (com), and overexpression (OErtfA) strains were point-inoculated onto GMM medium supplemented with 1.2 M sorbitol, 0.6 M KCl, or 1.0 M sucrose to induce osmotic stress. Plates were incubated at 37°C and colony diameters were measured at 48 h (A). Error bars represent standard error. (B) Photographs of colonies from 48 h of incubation. (PDF) [file pone.0176702.s010.pdf]

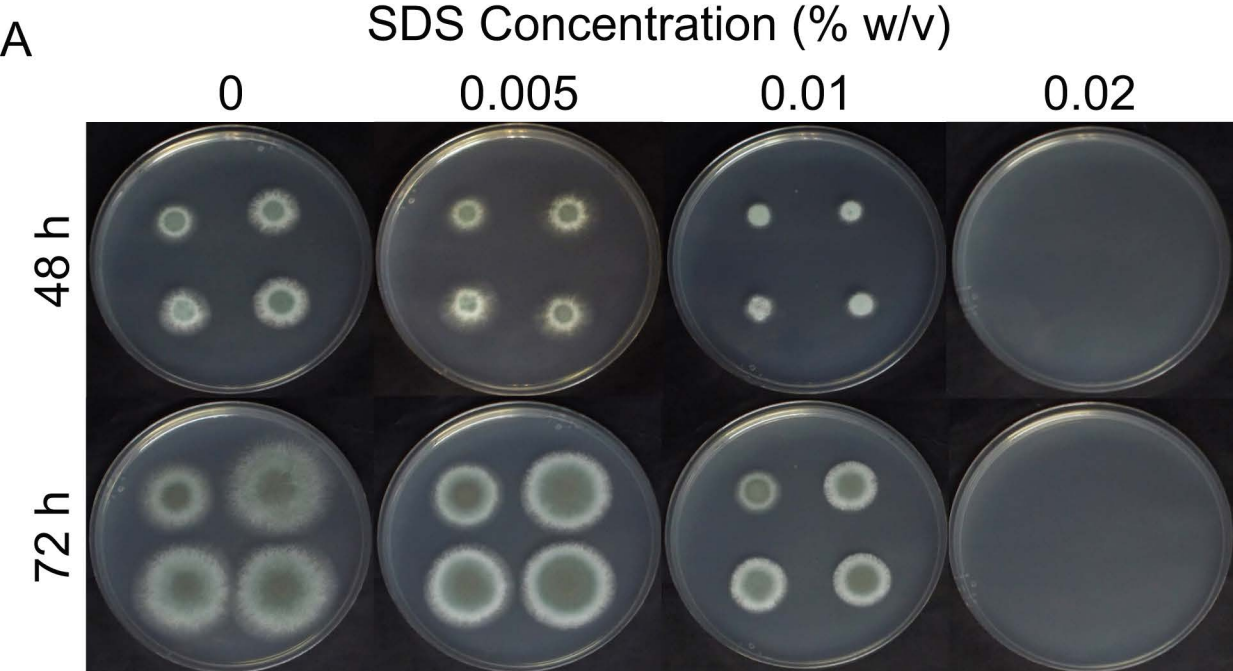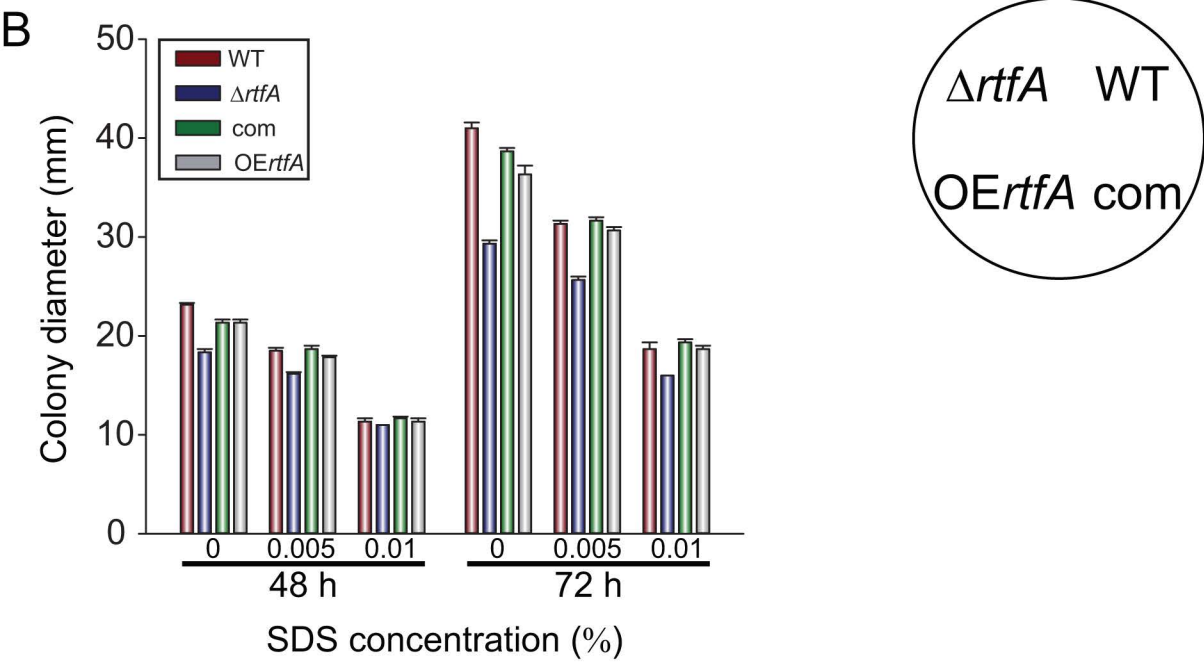

Supplement: S9 Fig — Wild type (WT), deletion (ΔrtfA), complementation (com), and overexpression (OErtfA) strains were point-inoculated on GMM supplemented with increasing concentrations of SDS. Strains were incubated at 37°C. (A) Photographs of colony at 48 h and 72 h. (B) Colony diameters measured at 48 h and 72 h after inoculation. Error bars represent standard error. (PDF) [file pone.0176702.s011.pdf]

24 h

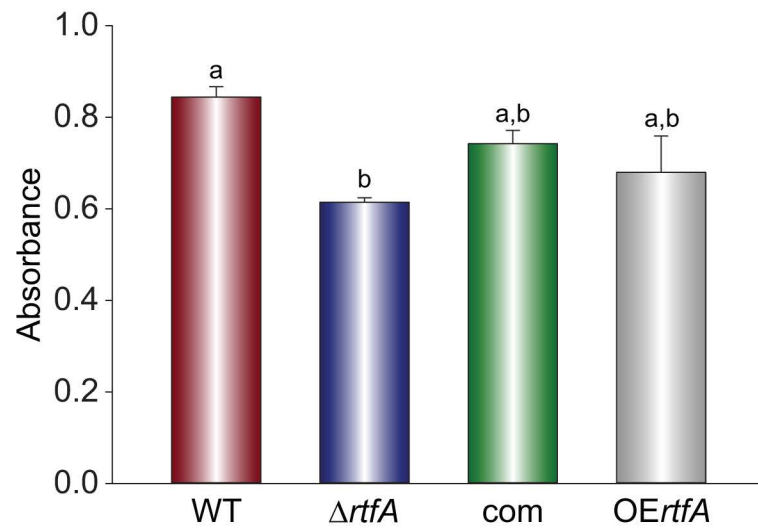

48 h

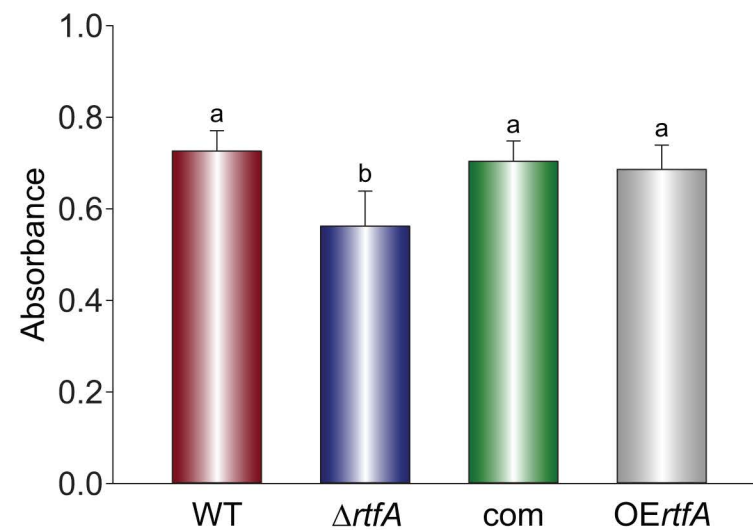

72 h

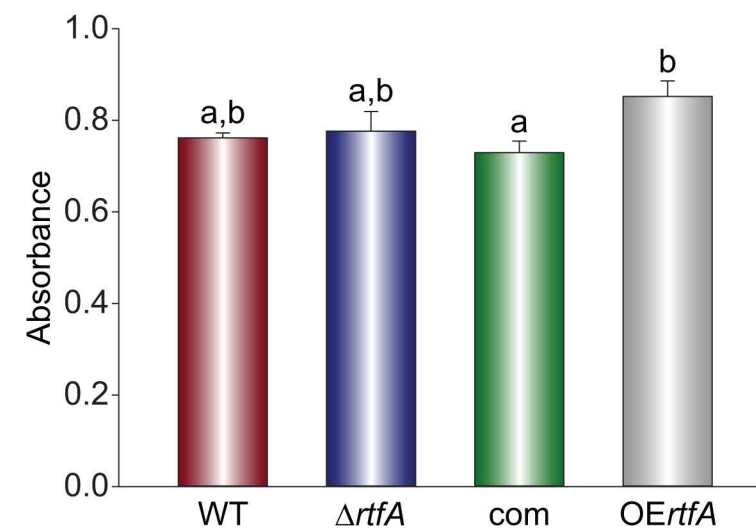

Supplement: S10 Fig — Strains were inoculated in liquid GMM and incubated at 37°C for 24 h (A), 48 h (B), and 72 h (C). After staining with Crystal Violet the absorbance was read at 560 nm. Samples were diluted 3 fold. Different letters indicate statistically different values (p ≤ 0.05). Error bars represent standard error. (PDF) [file pone.0176702.s012.pdf]
